# Supplementary material for: Deriving waveform parameters from calcium transients in human iPSC-derived cardiomyocytes to predict cardiac activity with machine learning
Source: Stem Cell Reports. 2022 Feb 10;17(3):556–68. doi: 10.1016/j.stemcr.2022.01.009 (PMC9039838; doi:10.1016/j.stemcr.2022.01.009)
Supplement: Document S1. Figures S1–S7 and Tables S1–S5 [file mmc1.pdf]

Stem Cell Reports, Volume *17*

Supplemental Information

Deriving waveform parameters from calcium transients in human iPSC-derived cardiomyocytes to predict cardiac activity with machine learning

Hongbin Yang, Will Stebbeds, Jo Francis, Amy Pointon, Olga Obrezanova, Kylie A. Beattie, Peter Clements, James S. Harvey, Graham F. Smith, and Andreas Bender

Figure S1. Occurrence of multi-peak of the 10 compounds with the highest occurrence.

The blue bars indicate the number of samples of the compound, and the green bars are the number of samples where at least one multiple-peak occurs. Only Ivabradine shows a significantly high occurrence compared to the background of negative control samples ($p<0.05$). Though the others are not significant, it is still possible that the multi-peak results from the effect of the compounds rather than the inherent variety of hiPSC-CMs.

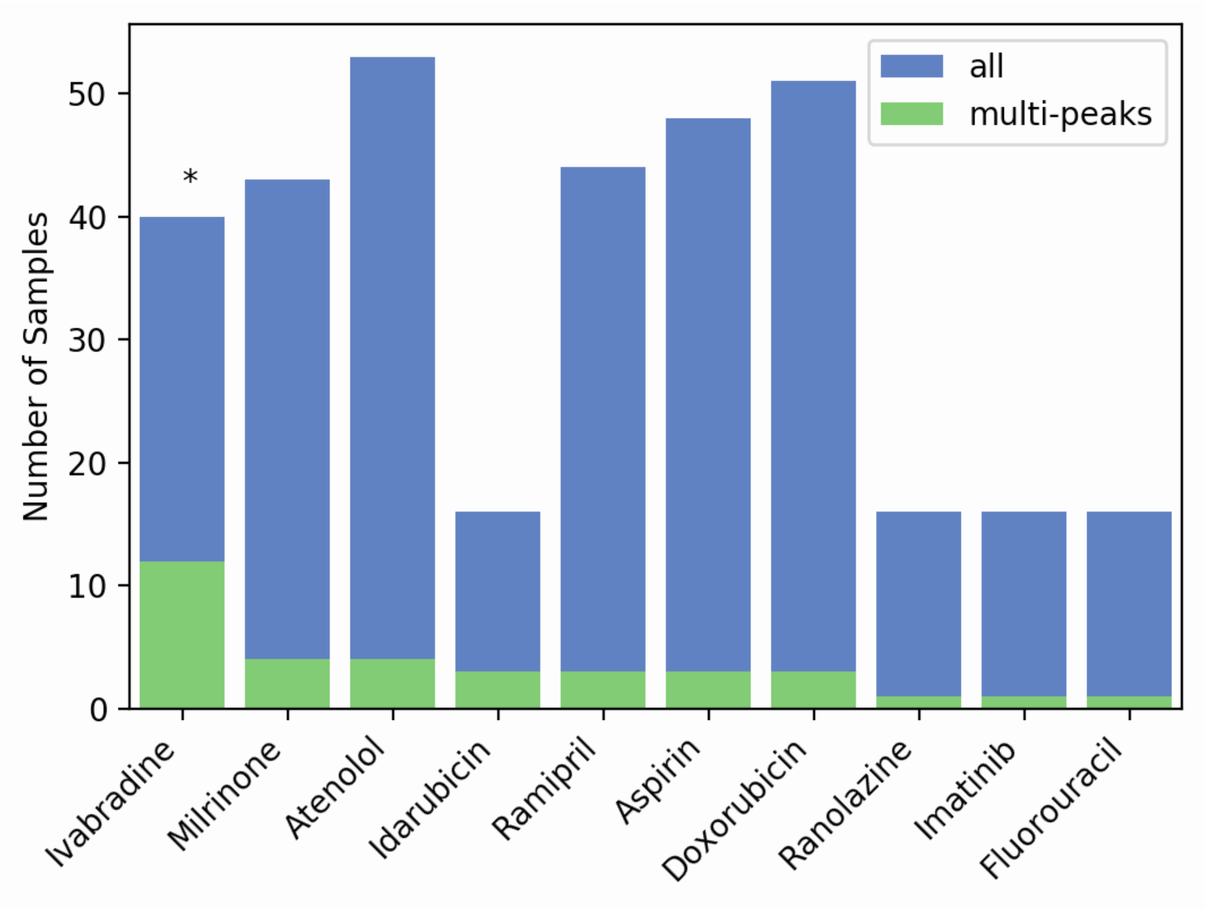

Figure S2. Samples with high Average Shoulder/Tail.

For replicates, only the highest Average Shoulder/Tail was kept and samples with Average Amplitude < 100 were removed since they are probably beat stop samples. It shows that almost all the samples with a high Average Shoulder/Tail (>0.5) are active, indicating that this parameter has very high precision in predicting cardiac activity. The only false positive, Buspirone, is also reasonable because it may have mild cytotoxicity under a high concentration (50 μM).

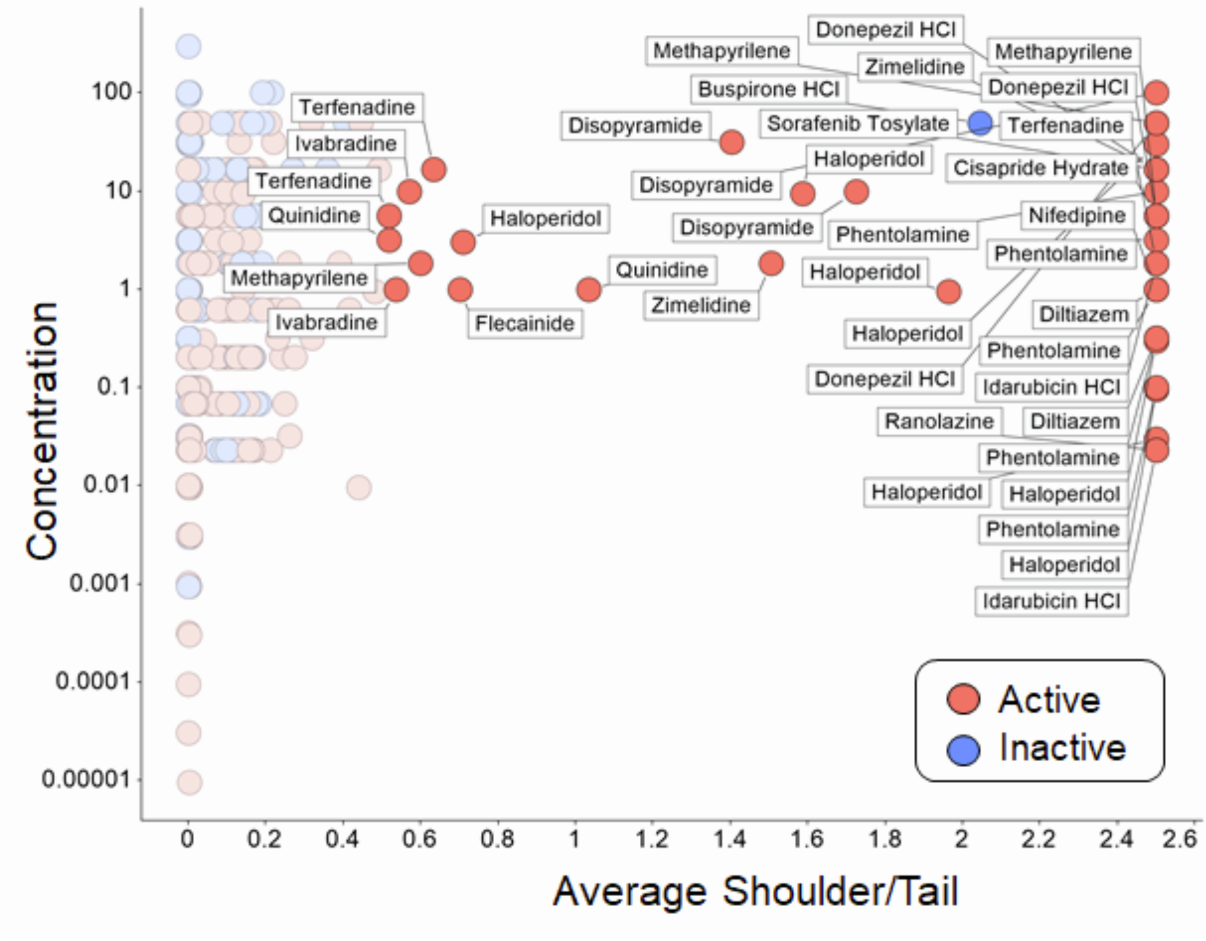

Figure S3. The sum of correlation coefficients between parameters and cardiac activities under different concentrations.

It can be seen that as the concentration increases, the correlation coefficients between the parameters and cardiac activity become higher in a general view.

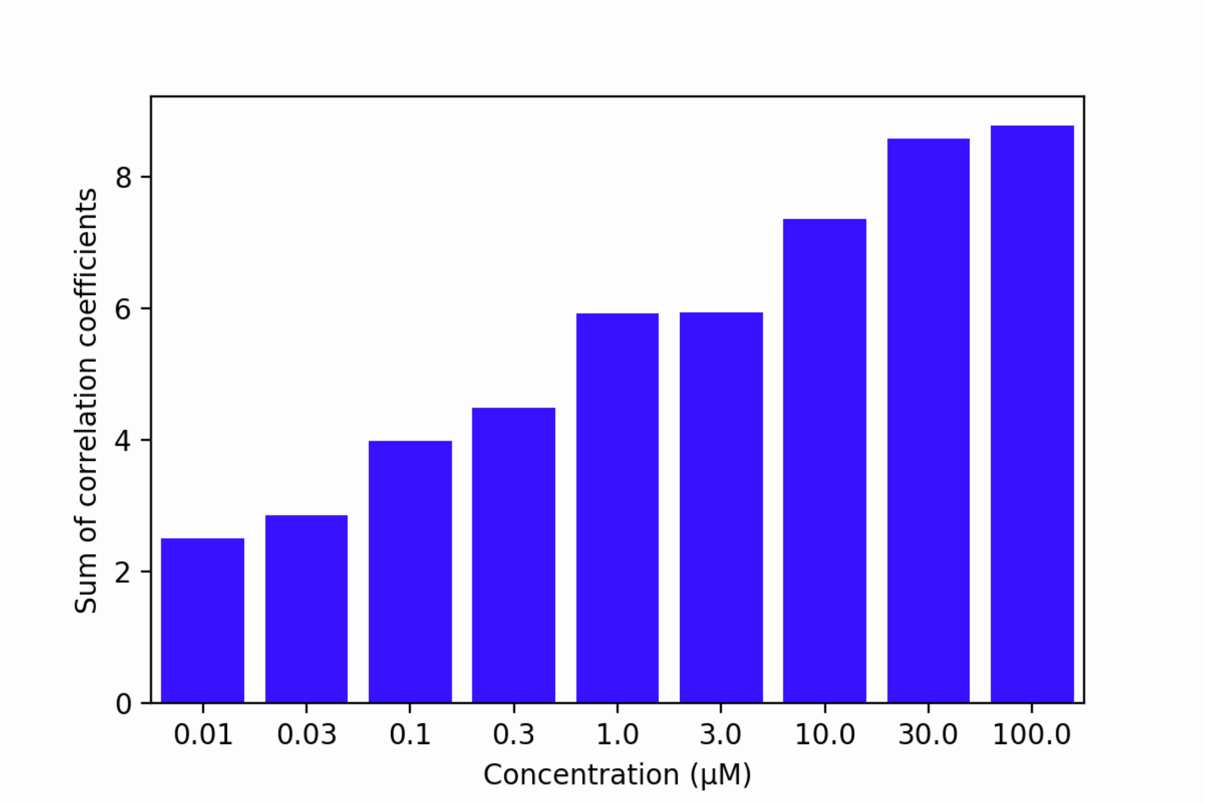

Figure S4. The Pearson coefficients between two parameters.

Generally, the coefficients are low except for some amplitude related parameters (RMS, Average Peak Amplitude and Average Intensity), indicating that these parameters can give much information to describe the waveform.

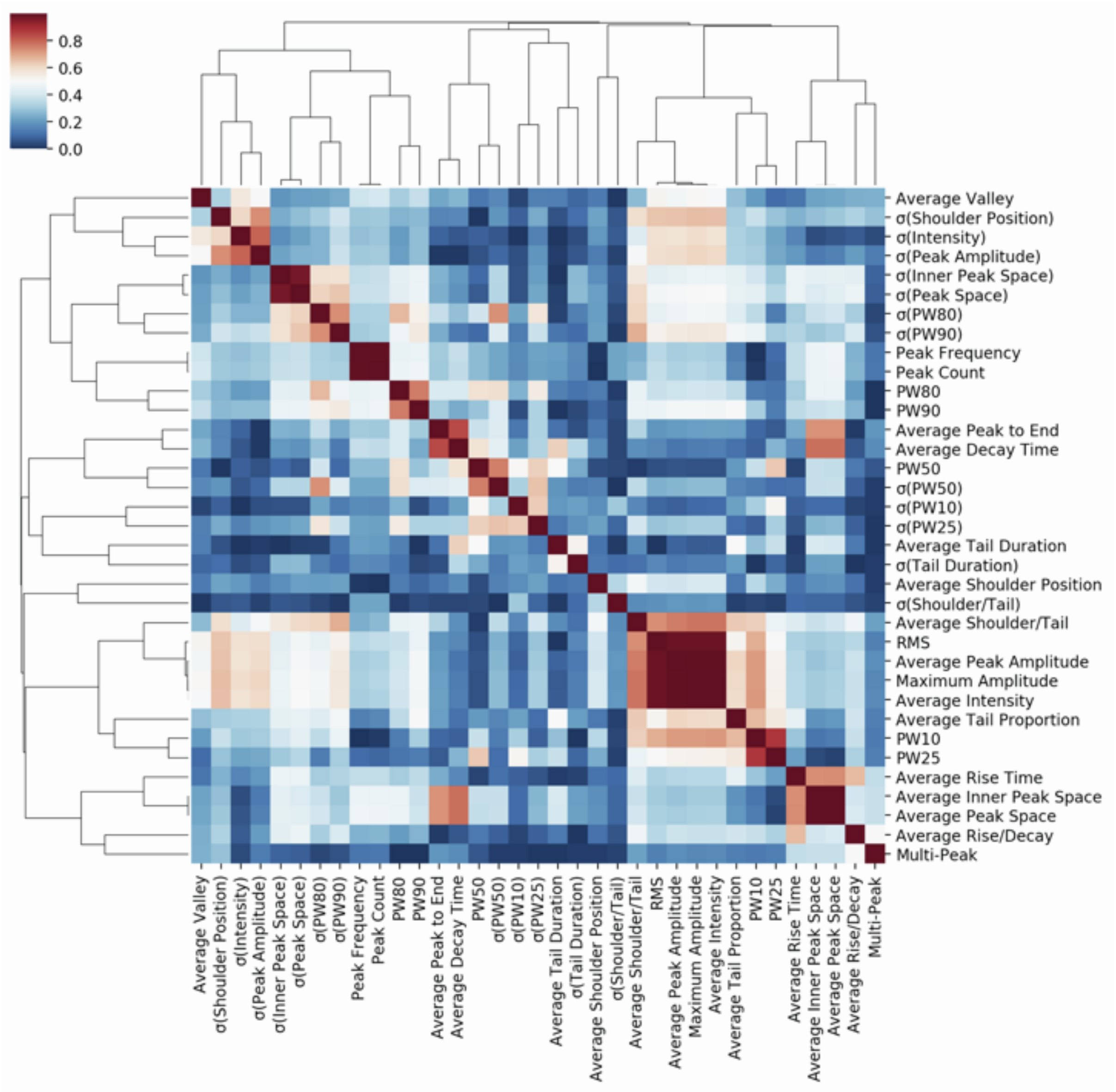

Figure S5. Distributions of the top 25 parameters ranked by the sum of the correlation between the parameters and the cardiac activity.

Samples are classified into three groups, vehicle (Neg) and positive (Pos) controls and other compounds (Treat). It can be seen that for most of the parameters, there is a clear difference between vehicle and negative controls and the treatment samples have overlapped with the positive and vehicle controls.

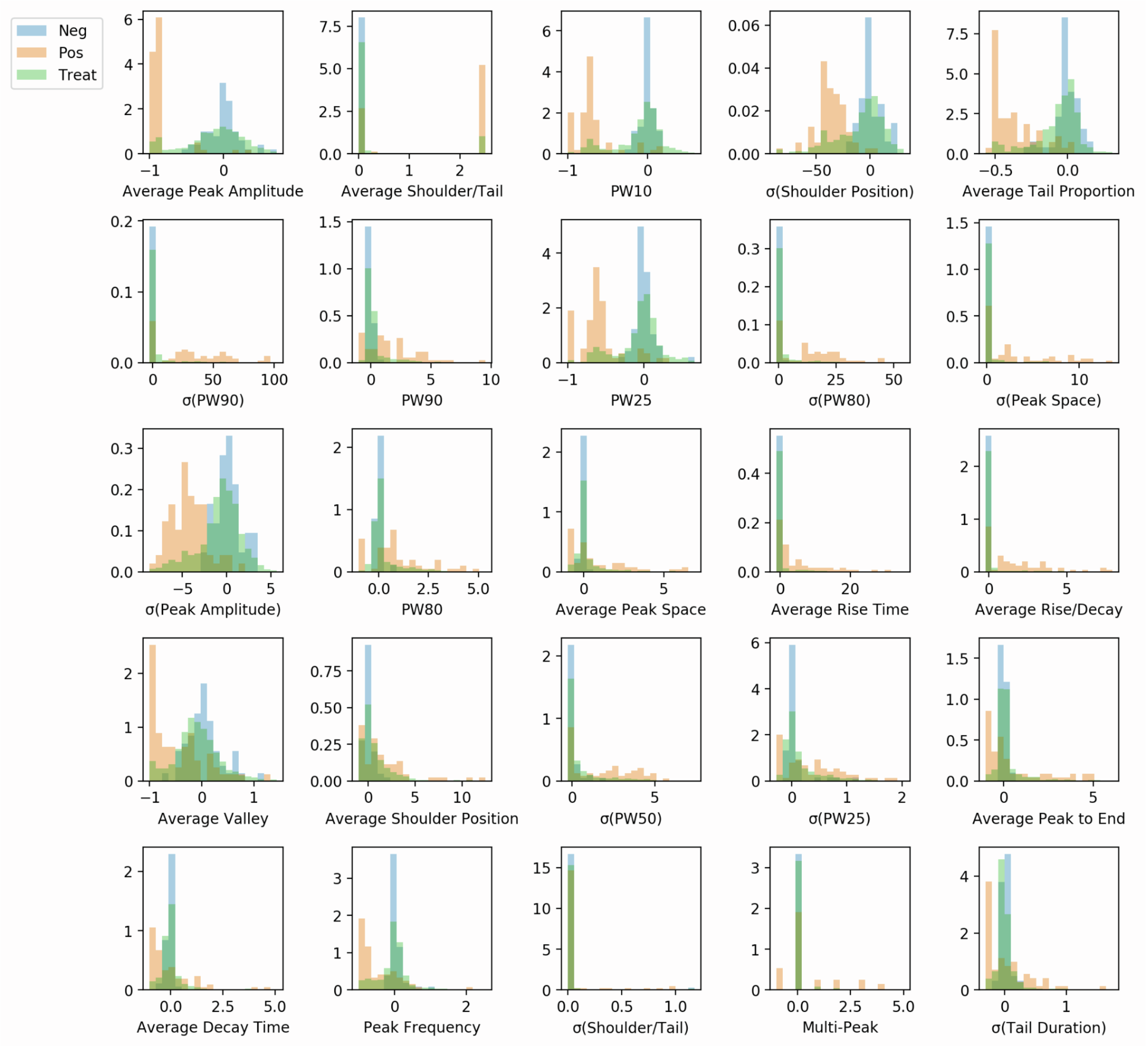

Figure S6. Principal component analysis of the top 25 parameters for active compounds.

The colour map is based on the logarithm of the concentrations, darker colours mean lower concentration. We can see that low concentration samples tend to be in the area which is overlapped with inactive compounds.

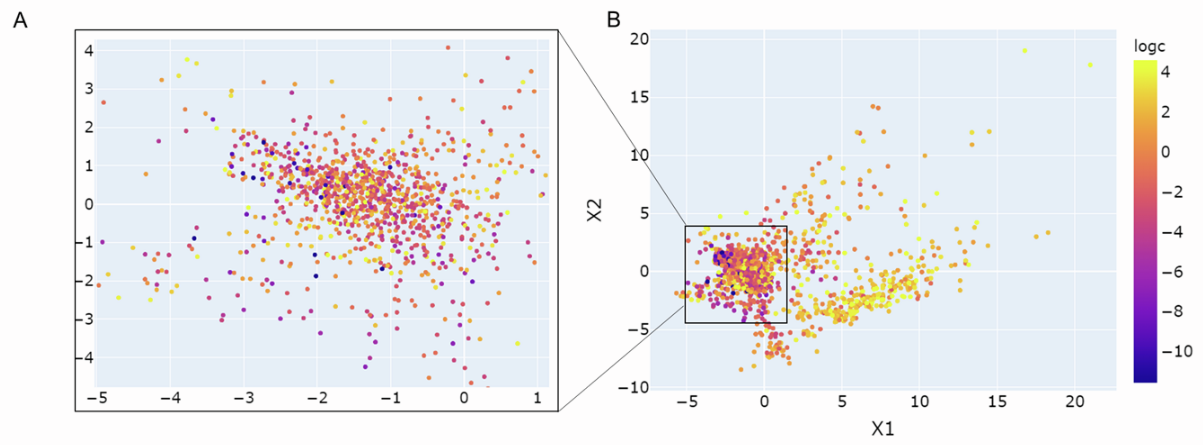

Figure S7. Distribution of distances between biological and technical replicates.

The distributions of the two datasets are almost the same to their mix, indicating that biological replicates and technical replicates present the same waveform varieties in terms of the parameters.

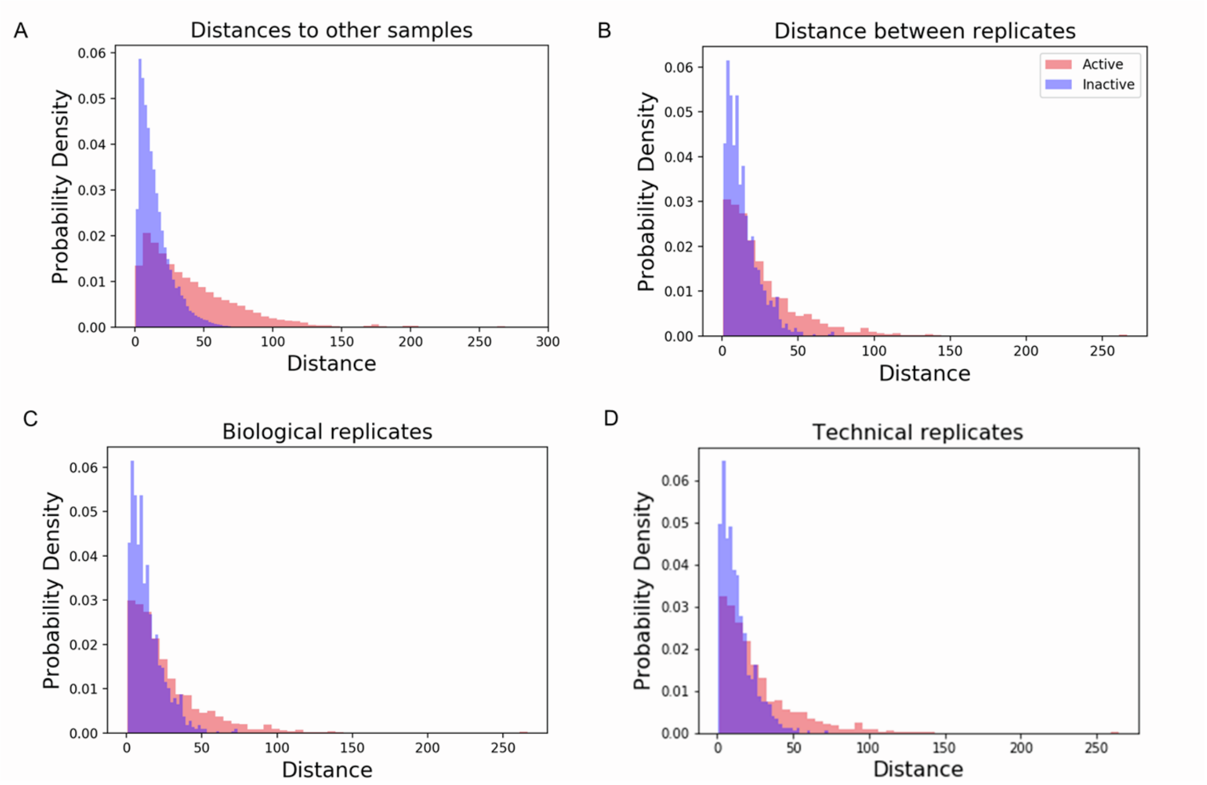

Table S1. Parameters that can be calculated by the toolkit.

Name	Variable	Type	Used in modelling	Annotation
Average Rise Time	up_length	Value	TRUE	Average rise time
Average Decay Time	down_length	Value	TRUE	Average duration of decreasing waveform, from peak point to the first point lower than valley value plus variance (10% of max)
Average Peak To End	full_down	Value	TRUE	Average of duration between peak and next starting point
Average Rise/Decay	rd_ratio	Ratio	TRUE	Average rise time/decay time ratio
Number of Peaks	n_peak	Value	FALSE	Number of peaks
Peak Frequency	freq	Value	TRUE	Number of period for the 100 seconds, 100/Peak Space
Maximum Amplitude	maximum	Value	FALSE	The highest amplitude
Average Peak Amplitude	avg_amplitude	Value	TRUE	Average peak amplitude
σ(Amplitude)	std_amplitude	Deviation	TRUE	The standard deviation of peak amplitudes
Average Intensity	avg_intensity	Value	FALSE	The average intensity (intensity is the highest signal in a cycle)
σ(Intensity)	std_intensity	Deviation	FALSE	The standard deviation of intensity
RMS	rms	Value	FALSE	Root mean square of the peak amplitudes
Average Peak Space	avg_lambda	Value	TRUE	The average duration of period
σ(Peak Space)	std_lambda	Deviation	TRUE	The standard deviation of period
Average Inner Peak Space	avg_inner_lambda	Value	FALSE	The average duration of periods removing outliers

σ(Inner Peak Space)	std_inner_lambda	Deviation	FALSE	The standard deviation of period removing outliers
Tail Proportion	tail_proportion	Ratio	TRUE	The average proportion of tail in one period
Average Tail Duration	avg_tail	Value	FALSE	The average duration of tail
Average Valley	avg_valley	Value	TRUE	The average intensity of valley points
Average Shoulder Position	avg_shoulder	Ratio	TRUE	The average relative position of shoulder in a period (0-1)
σ(Shoulder Position)	std_shoulder	Deviation	TRUE	The standard deviation of shoulder position
Average Shoulder/Tail	avg_shoulder_tail	Ratio	TRUE	The average ratio between prominence of shoulder and tail in a kernel density distribution
σ(Shoulder/Tail)	std_shoulder_tail	Deviation	TRUE	The standard deviation of shoulder tail ratio
Multi-Peaks	max_combo_peaks	Value	TRUE	Maximum of the number of peaks in one period
PWXX	PWXX_mean	Value	TRUE	The average of peak width at XX% from peak to bottom. XX is in {10, 25, 50, 80, 90}
σ(PWXX)	PWXX_std	Deviation	TRUE	Standard deviation of the peak widths
Uniformity	uniform	Binary	FALSE	Peak uniformity ($p > 0.99$ in KS test)
Noisy Waveform	noise	Binary	FALSE	If the waveform is noise, which means it is probabily a bad waveform
Double Peak	double_peak	Binary	FALSE	If there is double peak (max_combo_peaks > 1)
Fail	fail_analysis	Binary	FALSE	If there is a missing key point (starting point, down point etc.)

Used in modelling means whether the parameter(s) are used in machine learning models (25 in total)

Table S2. Numbers of samples and compounds used in this study

Data source	Compounds[1]	Test time point	Raw samples	Negative control samples	Positive control samples	Removed samples	Normalised samples
AstraZeneca	38	Baseline	2072	84	84	-	-
		0.5 hour	2072	59	69	390	1,554
GSK	36	1 hour	304	15	0	1	288
		72 hours	304	16	0	0	288
Sum	63		4752[2]	-	-	-	2,130

[1]Control compound (DMSO) is not counted in "compounds" column

[2]They are total number of waveforms, not the real samples

Table S3. Profile of the compounds

Compound name	Concentrations (μM)	Ground truth	Source	Duration
Amiodarone	0.023, 0.069, 0.206, 0.617, 1.852, 5.556, 16.667, 50	Active	GSK	1 hr, 72 hr
Amitriptyline	0.01, 0.03, 0.1, 0.3, 0.95, 3, 9.49, 30	Active	AZ	30min
Amoxicillin	0.1, 0.3, 0.95, 3.01, 9.51, 30.04, 94.94, 300	Inactive	AZ	30min
Amphotericin B	0.023, 0.069, 0.206, 0.617, 1.852, 5.556, 16.667, 50	Active	GSK	1 hr, 72 hr
Aspirin	0.03, 0.1, 0.32, 1.0, 3.17, 10.01, 31.65, 100	Inactive	AZ	30min
Atenolol	0.01, 0.03, 0.1, 0.3, 0.95, 3, 9.49, 30	Active	AZ	30min
Bepridil	0.003, 0.01, 0.03, 0.1, 0.32, 1.0, 3.16, 10	Active	AZ	30min
Buspirone	0.023, 0.069, 0.206, 0.617, 1.852, 5.556, 16.667, 50	Inactive	GSK	1 hr, 72 hr
Captopril	0.03, 0.1, 0.32, 1.0, 3.17, 10.01, 31.65, 100; 0.023, 0.069, 0.206, 0.617, 1.852, 5.556, 16.667, 50	Active	AZ/GSK	30min, 1 hr, 72 hr
Chloroquine	0.03, 0.1, 0.32, 1.0, 3.17, 10.01, 31.65, 100	Active	AZ	30min
Cibenzoline	0.03, 0.1, 0.32, 1.0, 3.17, 10.01, 31.65, 100	Active	AZ	30min
Cimetidine	0.03, 0.1, 0.32, 1.0, 3.17, 10.01, 31.65, 100	Inactive	AZ	30min
Cisapride	0.023, 0.069, 0.206, 0.617, 1.852, 5.556, 16.667, 50	Active	GSK	1 hr, 72 hr
Clonidine	0.01, 0.03, 0.1, 0.3, 0.95, 3, 9.49, 30	Active	AZ	30min
Clozapine	0.023, 0.069, 0.206, 0.617, 1.852, 5.556, 16.667, 50	Active	GSK	1 hr, 72 hr
Cyclophosphamide	0.023, 0.069, 0.206, 0.617, 1.852, 5.556, 16.667, 50	Inactive	GSK	1 hr, 72 hr
Cytochalasin D	0.023, 0.069, 0.206, 0.617, 1.852, 5.556, 16.667, 50	Active	GSK	1 hr, 72 hr
Digoxin	0.003, 0.01, 0.03, 0.1, 0.32, 1.0, 3.16, 10	Active	AZ	30min
Diltiazem	0.03, 0.1, 0.32, 1.0, 3.17, 10.01, 31.65, 100	Active	AZ	30min
Disopyramide	0.03, 0.1, 0.32, 1.0, 3.17, 10.01, 31.65, 100	Active	AZ	30min
Dobutamine	0.003, 0.01, 0.03, 0.1, 0.32, 1.0, 3.16, 10	Active	AZ	30min
Donepezil	0.023, 0.069, 0.206, 0.617, 1.852, 5.556, 16.667, 50	Active	GSK	1 hr, 72 hr
Doxorubicin	0.03, 0.1, 0.32, 1.0, 3.17, 10.01, 31.65, 100; 0.023, 0.069, 0.206, 0.617, 1.852, 5.556, 16.667, 50	Active	AZ/GSK	30min, 1 hr, 72 hr
Enalapril	0.03, 0.1, 0.32, 1.0, 3.17, 10.01, 31.65, 100; 0.023, 0.069, 0.206, 0.617, 1.852, 5.556, 16.667, 50	Inactive	AZ/GSK	30min, 1 hr, 72 hr
Epinephrine	0.00032, 0.001, 0.00317, 0.01, 0.0317, 0.1, 0.3165, 1; 0.023, 0.069, 0.206, 0.617, 1.852, 5.556, 16.667, 50	Active	AZ/GSK	30min, 1 hr, 72 hr
Erlotinib	0.023, 0.069, 0.206, 0.617, 1.852, 5.556, 16.667, 50	Inactive	GSK	1 hr, 72 hr
Flecainide	0.03, 0.1, 0.32, 1.0, 3.17, 10.01, 31.65, 100	Active	AZ	30min
Fluorouracil	0.023, 0.069, 0.206, 0.617, 1.852, 5.556, 16.667, 50	Active	GSK	1 hr, 72 hr

Furosemide	0.03, 0.1, 0.32, 1.0, 3.17, 10.01, 31.65, 100	Inactive	AZ	30min
Gemfibrozil	0.023, 0.069, 0.206, 0.617, 1.852, 5.556, 16.667, 50	Inactive	GSK	1 hr, 72 hr
Glyburide	0.03, 0.1, 0.32, 1.0, 3.17, 10.01, 31.65, 100	Active	AZ	30min
Haloperidol	0.01, 0.03, 0.1, 0.3, 0.95, 3, 9.49, 30	Active	AZ	30min
Idarubicin	0.023, 0.069, 0.206, 0.617, 1.852, 5.556, 16.667, 50	Active	GSK	1 hr, 72 hr
Imatinib	0.023, 0.069, 0.206, 0.617, 1.852, 5.556, 16.667, 50	Active	GSK	1 hr, 72 hr
Isoprenaline	0.00032, 0.001, 0.00317, 0.01, 0.0317, 0.1, 0.3165, 1; 0.023, 0.069, 0.206, 0.617, 1.852, 5.556, 16.667, 50	Active	AZ/GSK	30min, 1 hr, 72 hr
Ivabradine	0.03, 0.1, 0.32, 1.0, 3.17, 10.01, 31.65, 100	Active	AZ	30min
Ketoprofen	0.023, 0.069, 0.206, 0.617, 1.852, 5.556, 16.667, 50	Inactive	GSK	1 hr, 72 hr
Lapatinib	0.03, 0.1, 0.32, 1.0, 3.17, 10.01, 31.65, 100	Active	AZ	30min
Levosimendan	0.003, 0.01, 0.03, 0.1, 0.32, 1.0, 3.16, 10	Active	AZ	30min
Lisinopril	0.00095, 0.003, 0.0095, 0.3, 0.95, 3	Inactive	AZ	30min
Mebendazole	0.023, 0.069, 0.206, 0.617, 1.852, 5.556, 16.667, 50	Inactive	GSK	1 hr, 72 hr
Methapyrilene	0.023, 0.069, 0.206, 0.617, 1.852, 5.556, 16.667, 50	Active	GSK	1 hr, 72 hr
Milrinone	0.03, 0.1, 0.32, 1.0, 3.17, 10.01, 31.65, 100	Active	AZ	30min
Minoxidil	0.023, 0.069, 0.206, 0.617, 1.852, 5.556, 16.667, 50	Active	GSK	1 hr, 72 hr
Mitoxantrone	0.023, 0.069, 0.206, 0.617, 1.852, 5.556, 16.667, 50	Active	GSK	1 hr, 72 hr
Nandrolone	0.03, 0.1, 0.32, 1.0, 3.17, 10.01, 31.65, 100	Active	AZ	30min
Naringenin	0.023, 0.069, 0.206, 0.617, 1.852, 5.556, 16.667, 50	Inactive	GSK	1 hr, 72 hr
Nifedipine	0.003, 0.01, 0.03, 0.1, 0.32, 1.0, 3.16, 10; 0.023, 0.069, 0.206, 0.617, 1.852, 5.556, 16.667, 50	Active	AZ/GSK	30min, 1 hr, 72 hr
Omecamtiv Mecarbil	0.023, 0.069, 0.206, 0.617, 1.852, 5.556, 16.667, 50	Active	GSK	1 hr, 72 hr
Acetaminophen	0.03, 0.1, 0.32, 1.0, 3.17, 10.01, 31.65, 100	Inactive	AZ	30min
Phentolamine	0.03, 0.1, 0.32, 1.0, 3.17, 10.01, 31.65, 100	Active	AZ	30min
Pravastatin	0.03, 0.1, 0.32, 1.0, 3.17, 10.01, 31.65, 100; 0.023, 0.069, 0.206, 0.617, 1.852, 5.556, 16.667, 50	Inactive	AZ/GSK	30min, 1 hr, 72 hr
Praziquantel	0.023, 0.069, 0.206, 0.617, 1.852, 5.556, 16.667, 50	Inactive	GSK	1 hr, 72 hr
Quinidine	0.03, 0.1, 0.32, 1.0, 3.17, 10.01, 31.65, 100	Active	AZ	30min
Ramipril	0.03, 0.1, 0.32, 1.0, 3.17, 10.01, 31.65, 100	Inactive	AZ	30min
Ranitidine	0.03, 0.1, 0.32, 1.0, 3.17, 10.01, 31.65, 100	Inactive	AZ	30min
Ranolazine	0.023, 0.069, 0.206, 0.617, 1.852, 5.556, 16.667, 50	Active	GSK	1 hr, 72 hr
Sildenafil	0.01, 0.03, 0.1, 0.3, 0.95, 3, 9.49, 30; 0.023, 0.069, 0.206, 0.617, 1.852, 5.556, 16.667, 50	Inactive	AZ/GSK	30min, 1 hr, 72 hr

Sorafenib	0.023, 0.069, 0.206, 0.617, 1.852, 5.556, 16.667, 50	Active	GSK	1 hr, 72 hr
Sunitinib	0.01, 0.03, 0.1, 0.3, 0.95, 3, 9.49, 30; 0.023, 0.069, 0.206, 0.617, 1.852, 5.556, 16.667, 50	Active	AZ/GSK	30min, 1 hr, 72 hr
Terfenadine	0.023, 0.069, 0.206, 0.617, 1.852, 5.556, 16.667, 50	Active	GSK	1 hr, 72 hr
Tolbutamide	0.03, 0.1, 0.32, 1.0, 3.17, 10.01, 31.65, 100; 0.023, 0.069, 0.206, 0.617, 1.852, 5.556, 16.667, 50	Inactive	AZ/GSK	30min, 1 hr, 72 hr
Verapamil	0.003, 0.01, 0.03, 0.1, 0.32, 1.0, 3.16, 10; 0.023, 0.069, 0.206, 0.617, 1.852, 5.556, 16.667, 50	Active	AZ/GSK	30min, 1 hr, 72 hr
Zimelidine	0.023, 0.069, 0.206, 0.617, 1.852, 5.556, 16.667, 50	Active	GSK	1 hr, 72 hr

Table S4. Inter-correlation between parameters

Concentration (μM)	0.01	0.03	0.1	0.3	1	3	10	30	100
Average Peak Amplitude	0.093	0.127	0.242	0.219	0.321	0.338	0.453	0.486	0.475
Average Intensity	0.090	0.124	0.241	0.217	0.318	0.332	0.453	0.483	0.471
RMS	0.084	0.118	0.237	0.218	0.309	0.329	0.451	0.479	0.471
Maximum Amplitude	0.086	0.120	0.238	0.219	0.317	0.331	0.451	0.481	0.470
Average Shoulder/Tail	0.082	0.121	0.193	0.237	0.272	0.328	0.365	0.409	0.440
PW10	0.188	0.220	0.277	0.238	0.301	0.295	0.351	0.424	0.419
σ(Shoulder Position)	0.094	0.111	0.204	0.220	0.320	0.328	0.351	0.379	0.379
Average Tail Proportion	0.014	0.044	0.040	0.150	0.171	0.221	0.230	0.310	0.378
σ(PW90)	0.071	0.089	0.131	0.163	0.183	0.195	0.230	0.258	0.302
PW90	0.003	0.003	0.087	0.132	0.190	0.209	0.240	0.257	0.299
PW25	0.156	0.192	0.225	0.186	0.242	0.223	0.282	0.320	0.288
σ(PW80)	0.071	0.090	0.101	0.152	0.190	0.214	0.230	0.290	0.288
σ(Peak Space)	0.075	0.064	0.047	0.112	0.127	0.178	0.231	0.241	0.286
σ(Intensity)	0.024	0.003	0.071	0.033	0.115	0.141	0.213	0.301	0.282
σ(Amplitude)	0.025	0.047	0.090	0.053	0.126	0.090	0.264	0.276	0.281
σ(Inner Peak Space)	0.072	0.069	0.042	0.118	0.125	0.174	0.221	0.224	0.272
PW80	0.007	0.001	0.067	0.115	0.208	0.187	0.219	0.253	0.270
Average Peak Space	0.068	0.046	0.097	0.130	0.162	0.142	0.161	0.233	0.254
Average Inner Peak Space	0.066	0.043	0.095	0.130	0.161	0.140	0.157	0.227	0.248
Rise Time	0.049	0.028	0.042	0.084	0.109	0.084	0.101	0.234	0.236
Rise/Decay	0.050	0.036	0.073	0.081	0.122	0.118	0.157	0.197	0.216
Valley	0.061	0.035	0.055	0.028	0.139	0.126	0.255	0.203	0.198
Average Shoulder Position	0.178	0.180	0.148	0.193	0.188	0.121	0.044	0.058	0.176
σ(PW50)	0.125	0.147	0.185	0.204	0.231	0.250	0.321	0.314	0.174
σ(PW25)	0.140	0.159	0.151	0.122	0.194	0.161	0.191	0.222	0.173
Average Peak to End	0.012	0.024	0.085	0.123	0.159	0.159	0.155	0.123	0.160
Peak Count	0.054	0.094	0.059	0.068	0.025	0.007	0.067	0.129	0.151
Decay Time	0.045	0.036	0.098	0.106	0.129	0.141	0.157	0.132	0.151
Peak Frequency	0.051	0.093	0.057	0.071	0.018	0.001	0.056	0.120	0.142
σ(Shoulder/Tail)	0.107	0.140	0.143	0.118	0.149	0.127	0.085	0.191	0.130

Multi-Peak	0.082	0.065	0.012	0.033	0.046	0.033	0.012	0.105	0.121
σ(Tail Duration)	0.059	0.057	0.047	0.091	0.070	0.087	0.096	0.115	0.113
Average Tail Duration	0.037	0.014	0.062	0.067	0.094	0.089	0.098	0.063	0.039
PW50	0.024	0.039	0.040	0.014	0.068	0.044	0.004	0.014	0.021
σ(PW10)	0.067	0.072	0.011	0.051	0.024	0.001	0.014	0.034	0.009

Table S5. Performance of the machine learning model using leave-one-compound-out cross-validation

All data	Precision	Recall	F1 score	Accuracy	AUC
Sample-wise	0.84	0.85	0.84	0.79	0.84
Compound-wise	0.86	0.86	0.86	0.81	0.86
Simple model	0.82	0.84	0.83	0.76	0.83
Fingerprint-based model	0.72	0.95	0.82	0.71	0.6
Removing Inconsistent compounds					
Conventional method	0.97	0.76	0.85	0.81	-
Machine learning	0.88	0.9	0.89	0.85	-

Samples-wise means each sample is a data point when calculating metrics. Compound-wise means each compound is regarded as a data point by averaging the probability of different samples as a final output. The simple model was built by Number of Peaks and Average Peak Amplitude and assessed compound-wisely. The fingerprint-based model was built by molecular fingerprints (ECFP4) and random forest. The conventional method is based on concentration-response analysis using peak frequency and peak amplitudes. Inconsistent predictions between GSK and AZ were removed. The machine learning model is the same as the compound-wise one, but inconsistent compounds were removed during evaluation for a fair comparison.
