# Supplementary material for: Deriving waveform parameters from calcium transients in human iPSC-derived cardiomyocytes to predict cardiac activity with machine learning
Source: Stem Cell Reports. 2022 Feb 10;17(3):556–68. doi: 10.1016/j.stemcr.2022.01.009 (PMC9039838; doi:10.1016/j.stemcr.2022.01.009)
Supplement: Document S2. Article plus supplemental information [file mmc2.pdf]

Stem Cell Reports
Article
OPEN ACCESS

Deriving waveform parameters from calcium transients in human iPSC-derived cardiomyocytes to predict cardiac activity with machine learning

Hongbin Yang,[1] Will Stebbeds,[2] Jo Francis,[2] Amy Pointon,[3] Olga Obrezanova,[4] Kylie A. Beattie,[5] Peter Clements,[5] James S. Harvey,[5] Graham F. Smith,[4] and Andreas Bender[1,4,*]
[1]Centre for Molecular Informatics, Department of Chemistry, University of Cambridge, Cambridge, UK
[2]GlaxoSmithKline R&D, Stevenage, UK
[3]Functional and Mechanistic Safety, Clinical Pharmacology & Safety Sciences, BioPharmaceuticals R&D, AstraZeneca, Cambridge, UK
[4]Imaging and Data Analytics, Clinical Pharmacology & Safety Sciences, BioPharmaceuticals R&D, AstraZeneca, Cambridge, UK
[5]GlaxoSmithKline R&D, Ware, UK
*Correspondence: ab454@cam.ac.uk
https://doi.org/10.1016/j.stemcr.2022.01.009

SUMMARY

Human induced pluripotent stem cell-derived cardiomyocytes have been established to detect dynamic calcium transients by fast kinetic fluorescence assays that provide insights into specific aspects of clinical cardiac activity. However, the precise derivation and use of waveform parameters to predict cardiac activity merit deeper investigation. In this study, we derived, evaluated, and applied 38 waveform parameters in a novel Python framework, including (among others) peak frequency, peak amplitude, peak widths, and a novel parameter, shoulder-tail ratio. We then trained a random forest model to predict cardiac activity based on the 25 parameters selected by correlation analysis. The area under the curve (AUC) obtained for leave-one-compound-out cross-validation was 0.86, thereby replicating the predictions of conventional methods and outperforming fingerprint-based methods by a large margin. This work demonstrates that machine learning is able to automate the assessment of cardiovascular liability from waveform data, reducing any risk of user-to-user variability and bias.

INTRODUCTION

Cardiovascular toxicity often results in delays in drug discovery and development and additional clinical monitoring (Cook et al., 2014). For the past 25 years, the focus of cardiotoxicity assessments has centered around human ether-a-go-go-related gene (hERG)-mediated QT prolongation as a predictor of pro-arrhythmia risk (Wiśniowska et al., 2014). However, cardiovascular toxicity can also be the result of changes in hemodynamics (i.e., heart rate, blood pressure, and cardiac contractility) and/or changes in cardiovascular pathology. These elements are undetected via the assessment of pro-arrhythmia (Mellor et al., 2011). For example, doxorubicin induces cardiotoxicity via a diverse range of mechanisms, including oxidative stress and intracellular calcium dysregulation (Octavia et al., 2012), which would not be identified in *in vitro* ion-channel assays. As a consequence, several *in vitro* approaches have been developed recently to assess the potential of new chemical entities (NCEs) to induce structural and functional changes in cardiomyocytes. Human induced pluripotent stem cell-derived cardiomyocytes (hiPSC-CMs) have in particular been widely used in recent years, as they present an opportunity to consistently generate normal patient- and disease-specific cell lines (Gintant et al., 2016; Hoekstra et al., 2012).

hiPSC-CMs have offered the opportunity to assess cardiac activity in human cells at a scale and cost amenable to early drug discovery, as they require less time and effort than primary cell preparation (Bedut et al., 2016; Pointon et al., 2015; Spencer et al., 2014). With respect to readouts, for example, the assessment of calcium transients in hiPSC-CMs detected by fast kinetic fluorescence imaging has provided a higher-throughput assessment of cardiac contractility (Sirenko et al., 2013). In addition, hiPSC-CMs express key ion channels, receptors, enzymes, and kinases of human cardiomyocytes, and have relative calcium transients similar to those from mice and rabbits (Magdy et al., 2018), which are all beneficial factors with respect to the *in vivo* relevance of this model system. Thus, detecting calcium transients in hiPSC-CMs makes it possible to estimate cardiac activity in a high-throughput manner and still with practical relevance to the *in vivo* situation in humans. The question that then remains is how to analyze the data generated in an informative and unbiased manner, and that is what the current work aims to answer.

In-house scripts or commercial software are normally used to derive parameters from calcium transients (Juhola et al., 2015). More recently, an open-source R package, SVMCaT, was developed that can process calcium transients and derive peak-level and cell-level parameters, which were further used for abnormality assessment with expert labels and machine learning techniques (Hwang et al., 2020). CalTrack, a MATLAB-based algorithm, was developed to measure calcium transients from image stack to waveform parameters (Psaras et al., 2021). It has been

 © 2022 The Author(s).
This is an open access article under the CC BY license (http://creativecommons.org/licenses/by/4.0/).

reported that the commonly used parameters, such as peak frequency/beat rate, peak amplitude, and peak width, are in many cases suitable for detecting some compounds that have a risk of cardiotoxicity (Sirenko et al., 2013). In addition, CTD90, defined as calcium transient duration at 90% of decay following the peak amplitude (Ahola et al., 2018), was reported to have a high correlation with early afterdepolarization (EAD) (Kopljar et al., 2018), and the ratio of peak decay time to peak rise time (D/R ratio) has been shown to correlate with the concentration-dependent effects of cardiotoxic compounds (Watanabe et al., 2017). However, the number of parameters used in previous studies is still limited, and the potential of using machine learning models to predict cardiac activity of compounds for early drug discovery automatically, based on the hiPSC-CMs data described above, has been little explored.

The current study hence hypothesized that a set of novel parameters derived from the calcium transients in hiPSC-CMs would provide improved and automated insight into the potential cardiac effect of a compound. In this study, we developed a Python toolkit, CardioWave, to derive 38 parameters from calcium transient waveforms of 63 unique compounds, using data provided by AstraZeneca (AZ) and GlaxoSmithKline (GSK). In addition to conventional parameters such as peak frequency and amplitude-related parameters, we defined and calculated for all compounds several novel parameters, such as shoulder-tail ratio (shoulder/tail) and the presence of multiple peaks. We also demonstrated that these parameters can be used in combination with machine learning models to flag potential cardiac activity later in the clinic with a performance comparable to alternative approaches, but while automating the process and removing individual bias from it. The Python toolkit can also be used to analyze other kinds of waveform data, such as contractility, and to generate models for endpoints other than the ones employed.

RESULTS

Derivation of waveform parameters

The derivation of waveform parameters had two principal parts (for implementation details see also the experimental procedures). First, peak detection was implemented to separate the waveforms into individual wave cycles, from which single wave-cycle parameters, such as duration and amplitude, were calculated. Subsequently, statistical parameters, such as mean, standard deviation, and maximum, were used to describe the whole waveform. The following paragraphs give the details on how the parameters were derived from each wave cycle.

For each cycle of a waveform, parameters can be derived according to the key time points, including the rising point (the first point of a cycle), peak point (maximum of a cycle), tail starting point (the first point lower to 10% of maximum during decay), and valley point (the lowest point in a cycle). As shown in Figure 1A, by calculating the duration between the key time points, several time-related parameters, including rise time, decay time, peak to end, and tail duration, as well as the whole wavelength, peak space, can be derived. These parameters were commonly used in previous studies on calcium transient analysis (Yang et al., 2016; Yu et al., 2012). We also calculated peak widths at different prominences, including 10% (PW10), 25% (PW25), 50% (PW50), 80% (PW80), and 90% (PW90). These parameters depict the shape of the peaks and are commonly used in other waveform studies such as electrocardiogram (ECG) analysis (Lu et al., 2008). In addition to frequency-related parameters, amplitude-related parameters were also derived from each cycle, including amplitude, intensity, and valley (Figure 1B). With the parameters above, indirect parameters were calculated, including the ratio between rise time and decay time (rise/decay) and the ratio between tail duration and peak space (tail proportion).

Univariate kernel density estimation, which was performed to derive shoulder-related parameters (Racine, 2008), was implemented by *statsmodels*, a Python module that provides classes and functions for the estimation of many different statistical models (Seabold and Perktold, 2010). The biweight kernel was used and the bandwidth was set to 0.2 times the peak amplitude. In the density distribution, the peak with a prominence (x axis) between 0.15 and 0.8 times the maximum was regarded as the shoulder, and that with a prominence higher than 0.8 times the maximum was regarded as the tail (Figures 1C and 1D). Then, shoulder position (ratio to the prominence of the peak) and the shoulder/tail were recorded as the parameters of this cycle. To avoid extremely high values when tail density was close to 0, the maximum shoulder/tail was set to 2.5.

To illustrate why we expected the above types of parameters to provide additional insight, Figure 1E shows three artificial peaks, which have the same peak amplitude and peak space but different shapes. Peak widths at different prominences were considered as a way to represent the shape and, in this example, these three peaks have similar peak widths at the top (10% prominence or PW10). The third peak, which has a shoulder at the middle, has a smaller PW25 but a similar PW80 compared with the other two. The first peak, which does not have a shoulder nor a long tail, has a higher PW90 compared with the other two. In our parameter derivation, we explicitly calculated the ratio between shoulder and tail based on density estimation. In the first waveform example, there is no obvious shoulder, and the tail is also small, so the shoulder/tail will be a small number (here 0.01). For the second example,

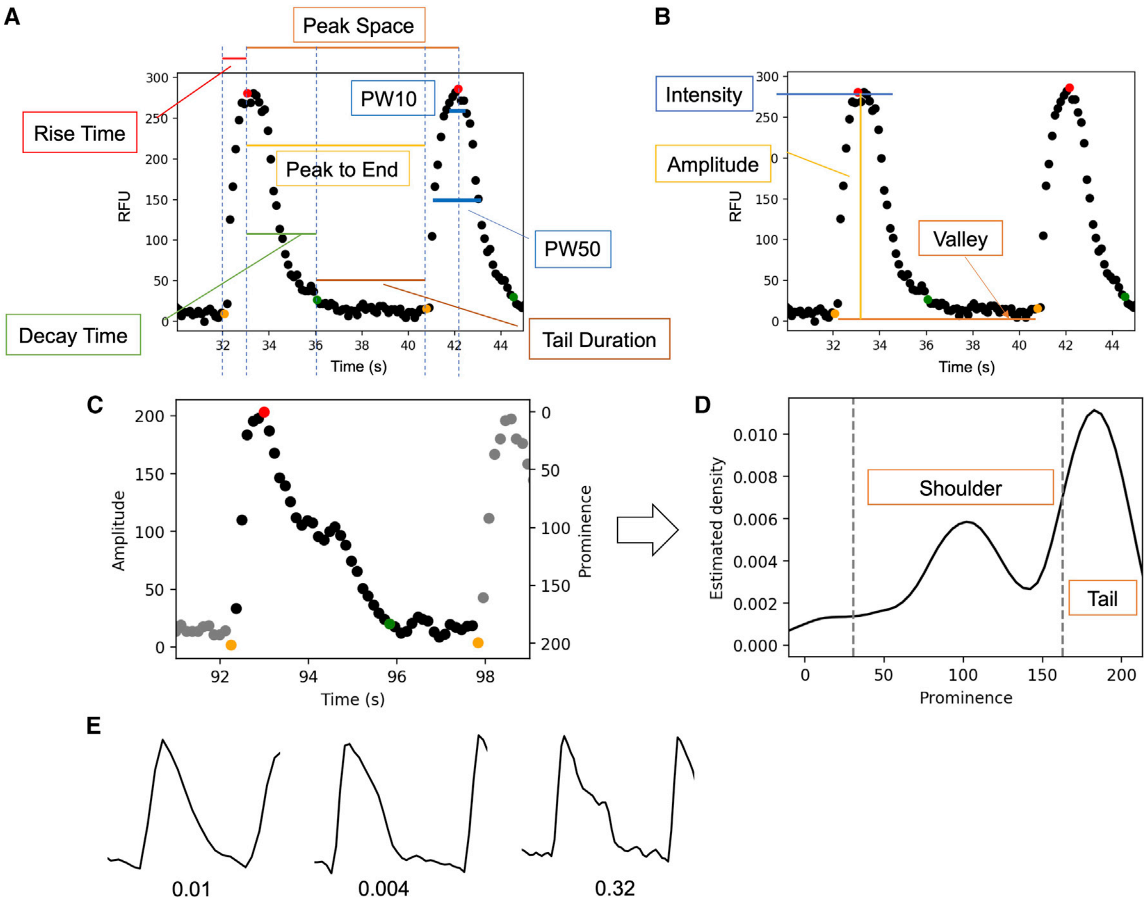

Figure 1. Frequency-, amplitude-, and shoulder-related parameters derived from each cycle in a waveform

(A) The definitions of rise time, peak to end, decay time, tail duration, and peak space. PW10 is the peak width at 10% of prominence from top to bottom (analogous to other peak-width measures).

(B) Definitions of amplitude, intensity, and valley. Amplitude equals the difference between intensity and valley.

(C) An example showing the shoulder/tail.

(D) The estimated density of amplitude of the waveform in (C).

(E) Synthetic waveform data with the same peak space but different shoulder and tail durations. It can be seen that the parameter added here is able to distinguish numerically between the different shapes shown.

where there is no shoulder but an obvious tail, the ratio will be close to 0 (here 0.004). In the last case, an obvious but small shoulder can be found (quantified numerically with a value of 0.32). With this parameter, we can characterize quantitatively the shapes of the peaks provided, without visual (manual) analysis.

A total of 38 parameters were derived from each waveform. These parameters were classified into four types: value, ratio, deviation, and binary (Table S1, which also includes the functions and parameters for analysis). Binary parameters were used only for checking the normality of waveforms and were not used in further analysis. For further details about the implementation of each parameter, see the document of the toolkit as well as the source code used, which accompanies this article.

Dataset, quality control, and normalization

Waveform data from two sources, namely AZ and GSK, were provided in slightly different formats, which required normalization steps to be performed before data merging. The dataset provided by AZ contained 39 compounds, tested 30 min after compound addition, with three parallel biological replicates and two technical replicates each (i.e., six replicates in total). In addition to the tested compounds, vehicle controls (0.1% DMSO) and positive controls, 10 μM verapamil, which will inhibit close to 100% of cardiomyocyte calcium transients (Harmer et al., 2012), were also included in this part of the data. For each sample from AZ, there is a corresponding baseline waveform, detected just before compound addition. The dataset provided by GSK contained 36 compounds and

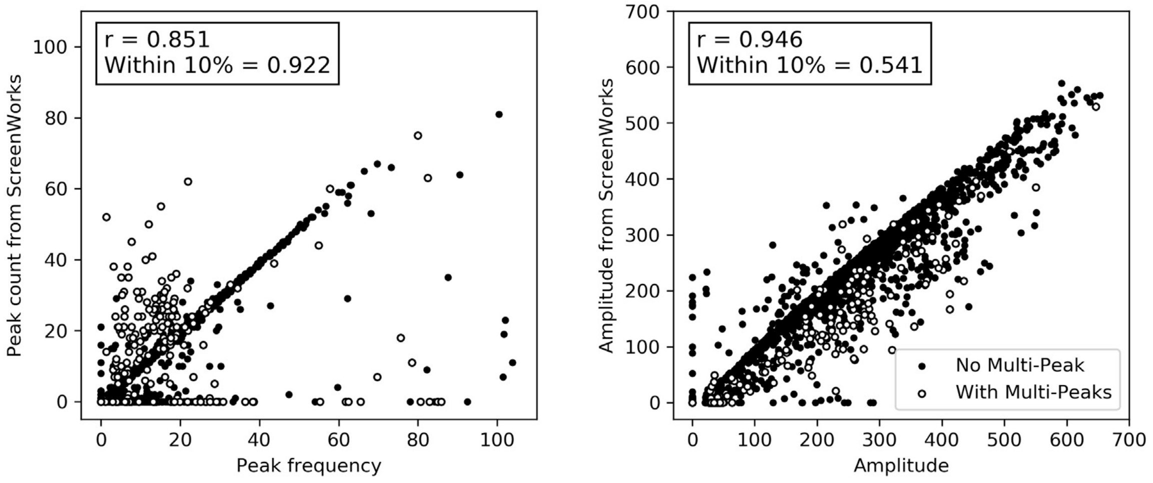

Figure 2. Comparison of the peak frequency and amplitude calculated in this study with those calculated by ScreenWorks
The "r" is the Pearson correlation coefficient of the samples excluding those with multi-peaks (black circles) removing zero points. "Within 10%" is the ratio of points where parameters derived by our toolkit are within 10% of the parameters derived from ScreenWorks.

vehicle controls (0.1% DMSO), but no positive controls. For each compound at a certain concentration, there were two samples provided, generated 1 and 72 h after compound addition, respectively. The samples from GSK were not replicated and baseline waveforms were not available.

After quality control and normalization (described in the experimental procedures), there were 2,130 compound-treated samples left from the original total of 2,680 samples. There were 69 zero-frequency samples removed, of which 28 were due to the low quality of baseline measurements. These included the waveforms with only one peak, which means the peak space is larger than the entire testing time (for which the parameters derived would be imprecise, as the peak is incomplete). In the remaining non-baseline dataset, 288 were observations following 72-h exposure, 288 were observations following 1-h exposure, and 1,554 were observations following 30-min exposure (Table S2). Compounds were ascribed a ground truth label of cardiac active or cardiac inactive, based on whether cardiac findings were mentioned in the FDA label of the compound (either a boxed warning, a warning and precaution, or an adverse reaction). Table S3 lists all the compounds with their cardiac activities, sources, and concentrations.

Plausibility analysis of derived parameters

To ensure the calculated parameters were plausible, the amplitude and peak frequency were compared with those calculated by ScreenWorks (Molecular Devices) for all compounds (version 3.2.0.14). The results (Figure 2) show that if we disregard the multi-peak waveforms and "zero parameters," the peak frequency and average peak amplitude calculated by this toolkit and ScreenWorks were very similar, with Pearson correlation coefficients of 0.851 and 0.946, respectively. The ratio of points that are within 10% of the difference is 0.922 and 0.541 for peak frequency and peak amplitude. Where there were discrepancies, the presence of multi-peaks was one of the major reasons leading to the inconsistency. ScreenWorks tended to regard a double peak as two peaks, so that the peak frequency would be higher than the value derived with the toolkit described here (Figure 2A).

The plausibility of using our additional novel parameters on real-world data was assessed next. The average intensity and standard deviation of peak space (σ(peak space)) are helpful to understand the shape of the waveforms as follows. Figure 3A shows samples of which the average intensity is lower than 100. It can be seen that their shapes are quite different in terms of regularity, and samples with a lower average amplitude tend to have high standard deviations. The two parameters can also help to quickly recognize abnormal waveforms based on their distribution. When σ(peak space) is very low, there is an obvious periodic variation in the waveform (Figure 3A, example 1), indicating a weak but regular calcium transient in cardiomyocytes. But for points like Figure 3A, example 2, the deviation is high, which means the signals are more likely noise from the detection platform. Figure 3A, example 3, is a waveform of which the calcium transients are not too weak but very irregular. Such waveforms have multi-peaks and will have a large deviation in the distance between two major peaks. They are not common over the whole dataset (only three samples), since most waveforms with high intensity have a low deviation of peak space.

We next analyzed the occurrence of multi-peaks. We found that multi-peaks are relatively common in our

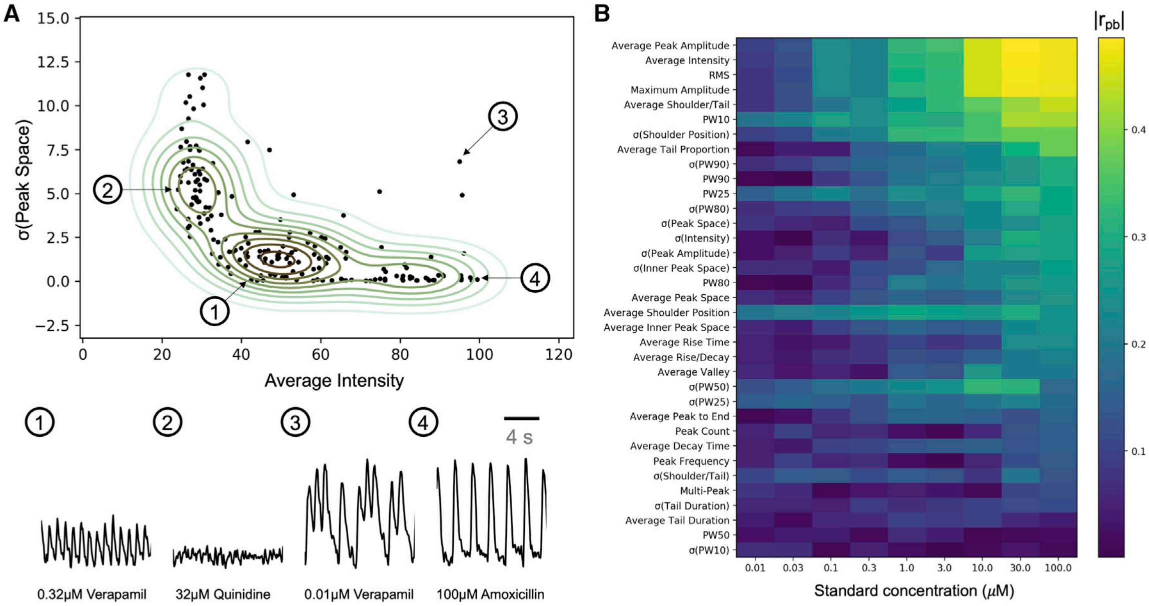

Figure 3. Parameter visualization and correlation to cardiac activity

(A) An illustration of how the deviation of peak space and average intensity is able to distinguish between more regular and less regular waveforms. The four examples are for visualization purposes only.

(B) The absolute point-biserial correlation coefficient $|r_{pb}|$ between parameters of waveforms at different concentrations and the cardiac activities of the compounds.

dataset: among the 116 vehicle controls there were 11 (9.4%) samples containing multi-peaks. The ratio was similar to the baseline waveforms (before adding compounds) and these wells had been removed during the quality control procedure. The analysis of the occurrence of multi-peaks of each compound showed that 20/63 compounds have at least one sample with multi-peaks. Figure S1 shows the 10 compounds with the highest multi-peak occurrence ratio. Ivabradine has shown a higher number of multi-peaks compared with the background of vehicle controls ($p = 0.013$), indicating that this compound may affect the cardiomyocytes via a specific mechanism that is different from that of other compounds. In the following analysis, ivabradine was removed because parameters such as frequency and decay time would have been imprecise and far different from the others in our dataset.

Another novel parameter developed in this study is the shoulder-tail ratio, namely shoulder/tail. Figure S2 plots all the compounds with their shoulder/tail and their concentrations. From this plot, most samples have a low shoulder-tail ratio, and for the samples with this parameter higher than 0.5, corresponding to 18 compounds, all are cardiac active except buspirone, which has a high shoulder-tail ratio at the concentration of 50 μM. The high precision of this parameter with cardiac activity illustrates the utility of one of our new waveform parameters for the prediction of cardiac activity.

Correlation between waveform-derived parameters and cardiac activity

We next evaluated the ability of individual waveform-derived parameters to anticipate cardiac activity across the full concentration range using correlation as a metric. The point-biserial correlation coefficients between the parameters and the ground truth labels are shown in Figure 3B (with numerical values being provided in Table S4). It can be seen that the correlation becomes higher when the concentration increases in terms of the sum of correlation coefficients, and the correlation is close to 0 when concentration is low (for more details see Figure S3). In particular, for the upper part of the plot, a good concentration-response relationship between parameters and cardiac activity label can be observed. This is generally the desired behavior, since gradual concentration-response curves provide significant benefits for later decision-making (such as deriving cutoff points and the like). As an exception, parameters such as PW10 and PW25 have relatively high correlation at low concentrations, indicating that these parameters might be sensitive to cardiac activity even in low concentrations.

The parameters with the highest correlation with the cardiac activity label are root-mean-square of signals (RMS), average amplitude, average intensity, maximum amplitude, shoulder/tail, and PW10, of which the correlation coefficients are more than 0.4. Peak frequency was

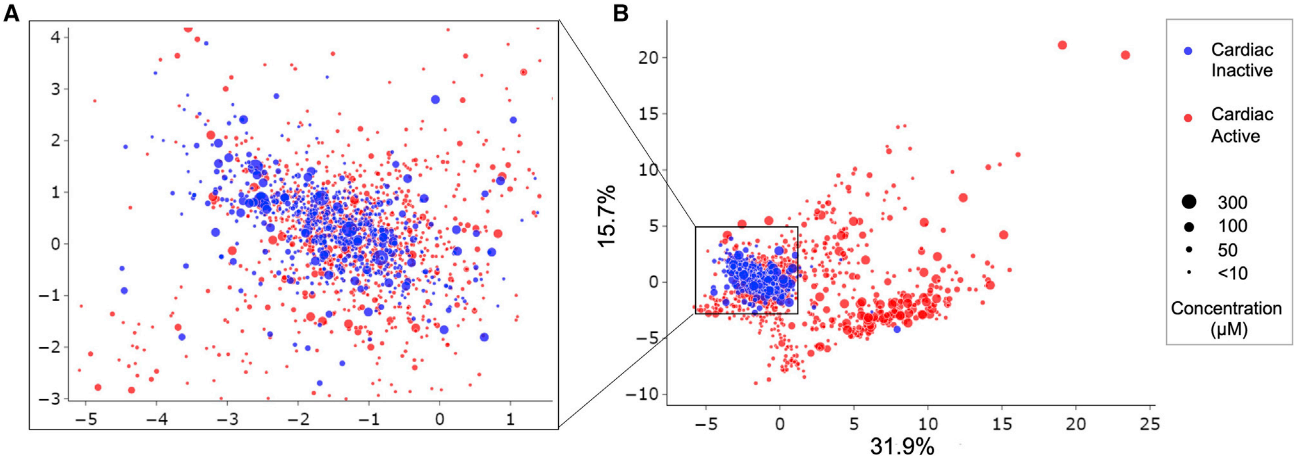

Figure 4. Principal-component analysis of the parameters

(A and B) Data points are sized by concentration. (A) shows the center of origin in (B) at increased resolution. The numbers on the axes of (B) are the percentage of variance explained by the first (x axis) and the second (y axis) principal components.

supposed to be one of the most important features in signaling analysis as it is related to the beat rate of the cardiomyocytes and is commonly used in relevant studies. However, in terms of the correlation shown in Figure 3B, the peak frequency-related parameters, including peak space, rise time, decay time, and peak to tail, which ranked 18, 20, 28, and 26 out of the 35 parameters, respectively, have far lower correlations than the amplitude-related parameters. Interestingly, standard deviation values such as σ(intensity) and σ(peak space) have a high correlation with cardiac activity, indicating also that the stability of the resulting waveform is related to cardiac activity.

Some parameters may be redundant, as their definitions are similar. To select the most information-rich parameters for subsequent modeling, we next calculated the correlation between the derived waveform parameters. The resulting heatmap (Figure S4) shows that these parameters are generally independent of each other, as their correlation coefficients are low. However, there are some parameter pairs that are highly correlated with each other, such as the amplitude-related parameters, maximum amplitude, intensity, and (average) amplitude. This is not unexpected, as these parameters were similarly defined and their values were almost identical for most waveforms, which also explains the similar patterns in the correlation heatmap (Figure 3B). Based on the intercorrelation, some redundant parameters were removed, including maximum amplitude, intensity, peak count, and inner peak space. In a final step, 25 parameters were selected based on their average correlation coefficients to the ground truth after removing the redundant parameters (see Table S1 for details of the parameters selected).

Distribution of the parameters between active and inactive compounds

We next tried to understand the distribution of the parameters and hence performed principal-component analysis (PCA) to compare visually the parameter distribution between cardiac- active and inactive compounds (Figure 4). This analysis shows that cardiac-inactive compounds tend to be clustered together, and they are overlapping with active compounds at lower concentrations (Figure S6). In Figure 4B, we can also discover some false positives (cardiac-inactive compounds predicted to be active). For example, buspirone is a representative false-positive compound. In addition, the active samples have different paths outward from the "inactive cluster" with increasing concentration tested, which implies that cardiac activity may have different modes of action (MOAs) in terms of the change of waveform.

The individual distributions of the 25 parameters selected from the previous section are shown in Figure S5. The overlap between active compounds and inactive compounds is small, and most samples are close to vehicle controls, which correspond to the inactive cluster of the PCA plot (Figure 4). In terms of the overlap area between positive control and vehicle control, the distribution of the parameters is consistent with the correlation analysis, and hence it can be concluded that the 25 parameters are potentially good features to describe calcium transients and to predict cardiac activities of these compounds at this stage of the analysis.

Replicate analysis and impact of concentration

To further understand the impact of concentration on the data generated, as well as data consistency, the distribution

of Euclidean distances between non-replicates was plotted, i.e., two compounds or the same compound but at different concentrations and between replicates (Figures S7A and S7B). The results show that for inactive compounds, their distances between non-replicates and between replicates were similar, ranging from 0 to 50, indicating that in the readouts used there was not more signal to distinguish compounds than there was to distinguish replicates. This is understandable, as they are inactive compounds, which are not meant to produce a signal with respect to the endpoints considered here. However, the distances of active compounds ranged from 0 to 150, indicating a higher variety of parameter values of active hiPSC-CMs. The non-replicate distances were generally higher than replicate distances for active compounds, implying that compounds had concentration-response behaviors as well as potentially different modes of action leading to different directions of waveform change, which is mentioned above. We also separated the replicate pairs into biological and technical replicates, and it shows that their distributions were almost the same as the mix of all replicates (Figures S7C and S7D). This means that biological replicates were as consistent as technical replicates in the readout analyzed here, allowing us to pool both types of replicates in the analysis.

Utilization of waveform-derived parameters for cardiac activity prediction

We next aimed to use the parameters derived from waveform data to predict cardiac activities of compounds, to gauge whether an automated analysis pipeline is feasible for automatic (and unbiased) data analysis. To this end, we obtained 303 samples across the 63 compounds by selecting 100 μM as the standard concentration, given that this concentration had the best correlation to cardiac activity in a previous analysis (Figure 3). It should be noted that for many compounds we do not have such high-concentration data available, and in those cases, the highest tested concentration has been used (Table S3). This step represented a limitation of the data available in the current study. We employed leave-one-compound-out cross-validation (LOCO-CV) to evaluate the random forest model built with the waveform-derived parameters. LOCO-CV was used due to the small sample size, and it should be seen more as an estimator of model consistency than a true estimate of its ability to predict the output variable for novel compounds (Hawkins, 2004). Considering the different sample sizes for different compounds, we evaluated model performance in both a compound-wise and a sample-wise manner. The precision and recall of the calcium transient-based model was 0.86, and the overall accuracy was 0.81 (Table S5). We can see the very low performance of the fingerprint-based model, as the accuracy was only 0.71, and the area under the curve (AUC) of this model was only 0.60, far lower than the calcium transient-based model, of which the AUC was 0.86 (Figure 5). By comparing the performance of the calcium transient-based and the fingerprint-based models, we can conclude that the parameters derived by calcium transients are a rather predictive feature set for cardiac activity of compounds based on the dataset used here. We also investigated the improvement from the added number of parameters derived using our approach against that of using standard features alone, namely, number of peaks and average peak amplitude, derived from the waveforms. It can be seen that our model with more parameters outperformed the simple model in all the metrics used (average accuracy of 0.81 versus 0.76, AUC of 0.86 versus 0.81, and F1 score of 0.86 versus 0.83). Hence, we can conclude that comprehensive features derived from the waveform perform best for detection of cardiac activity on the dataset used here, followed by simple waveform parameters, and that fingerprint-based models perform worst, in line with the hypothesis of this study.

The receiver operating characteristic (ROC) curve (Figure 5) of calcium transient-based models is sharp at the beginning, while the slope reduces dramatically when the true positive rate is around 0.65, indicating that in our dataset, more than half of the active compounds are readily predicted with few false positives, but it becomes more difficult to identify the other compounds. This can also be found in the predicted probabilities (means of replicates) of the individual compounds, shown in Figure 6, and indicates that subsets of compounds exist where our readouts more readily identify cardiac activity. This is likely related to a relatively consistent mode (or modes) of action of the compounds in the subset, which is more visible in the calcium transient readout employed here. The cardiac-active compounds correctly identified include not only known ion-channel blockers associated with QT prolongation and torsades de pointes (TdP) risk, such as sunitinib, but also compounds with inotropic effects, such as digoxin, a Na^+/K^+ ATPase inhibitor, indicating that calcium transients of hiPSC-CMs are indeed a surrogate for prediction of cardiac activity with different mechanisms.

On the other hand, some other modes of action also escaped the calcium transient readouts employed here, such as those of milrinone, atenolol, and captopril. The absence of response in the *in vitro* hiPSC-CMs assay for the endpoints served *in vivo* is related to the context of the assay platform and will be described in more detail in the discussion. Likewise, false-negative (cardiac-active compounds predicted to be inactive) predictions of cardiac activity were also influenced by the duration exposure of the test compound within the *in vitro* hiPSC-CMs and the subsequent effects on the calcium transients. One such

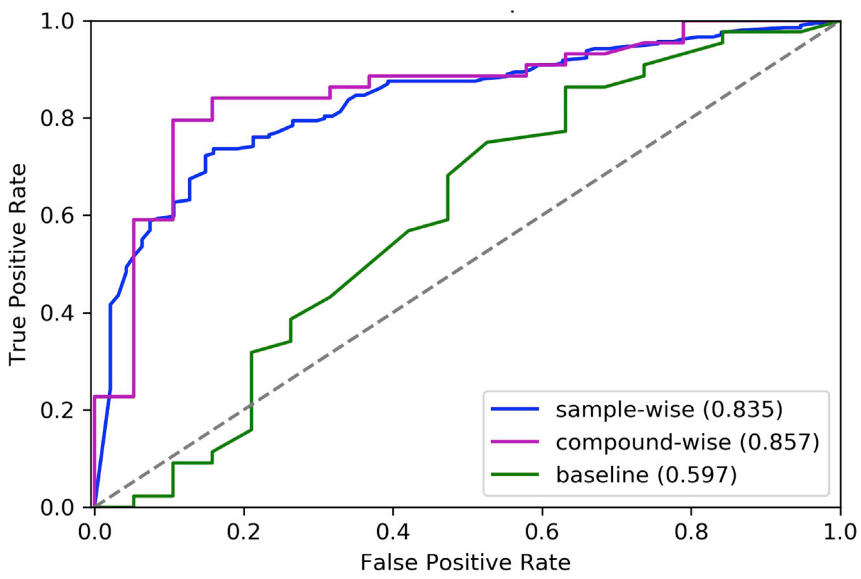

Figure 5. Receiver operating characteristic (ROC) curve of leave-one-compound-out random forest models

The final model was built with 38 waveform parameters, while the simple model used only two: number of peaks and average peak amplitude. All three models were evaluated compound-wise.

example is doxorubicin, a compound where the mechanisms of toxicity are complicated and remain incompletely understood. The results for doxorubicin indicated that over a short exposure period (30 min), only one sample (of six) had an obvious increase in amplitude; none of the other samples showed expected changes such as peak frequency, and this compound was predicted to be cardiac inactive. Over a longer exposure period (3 days), however, an obvious decrease in amplitude was observed, with beat stop occurring at the highest concentration, and therefore it was predicted to be cardiac active with a probability close to 1 (Figure 6). Although overall doxorubicin was predicted as a false negative under the averaging approach, which was biased by having more acute data available in the model, the waveforms did show changes in some cases, indicating also that the precise setup of the model, and drawing conclusions from changes in waveforms, requires careful adjustment to be useful in practice. In contrast, sildenafil was predicted with different output labels between long-term and short-term samples (Figure 6). However, because multiple short-term samples were predicted to be cardiac active and only one long-term sample was predicted to be inactive, it was finally falsely predicted as active. This also indicates that the utilization of short-term versus long-term time points deserves further fine-tuning in the future with respect to their predictive values.

To illustrate the predictivity of the machine learning models, we also compared the performance of the conventional methods used by GSK and AZ, which are based on concentration response of peak amplitude and peak count. When comparing these two methods, we removed inconsistent annotations from both sources (namely, captopril, sildenafil, and doxorubicin). These compounds were also removed from the machine learning model used for comparison. The overall accuracy of the predictions using conventional curve fitting was 0.81, slightly lower than the machine learning model (0.85). Compared with the conventional method, the machine learning model had a higher sensitivity (0.9 versus 0.76), but the precision was lower (0.88 versus 0.97, Table S5). Hence, we can conclude that the parameters and models derived in this work slightly outperform conventional methods for waveform classification (at a different precision/recall trade-off), while being able to automate unbiased compound labeling from the input data.

DISCUSSION

Using *in vitro* assays in combination with *in silico* models to replace the conventional animal-based assessment of *in vivo* cardiac activity is becoming increasingly important, given numerous ethical, scientific, business, and legislative incentives (Burden et al., 2015). Many studies have shown the usefulness of calcium transients in hiPSC-CMs as a high-throughput technique to detect the potential risk of cardiovascular toxicity by assessment of drug-induced long QT and TdP risk or cardiomyocyte contraction (Kopljar et al., 2018; Lu et al., 2019; Pointon et al., 2015). In this work, a Python toolkit was developed to derive parameters from hiPSC-CM calcium transient data for a set of exemplar compounds, which was followed by a statistical analysis to evaluate their relationship to the reported cardiac activity of compounds. Finally, *in vitro* calcium transient data and machine learning models were used in combination to evaluate the potential cardiac activities of the compounds

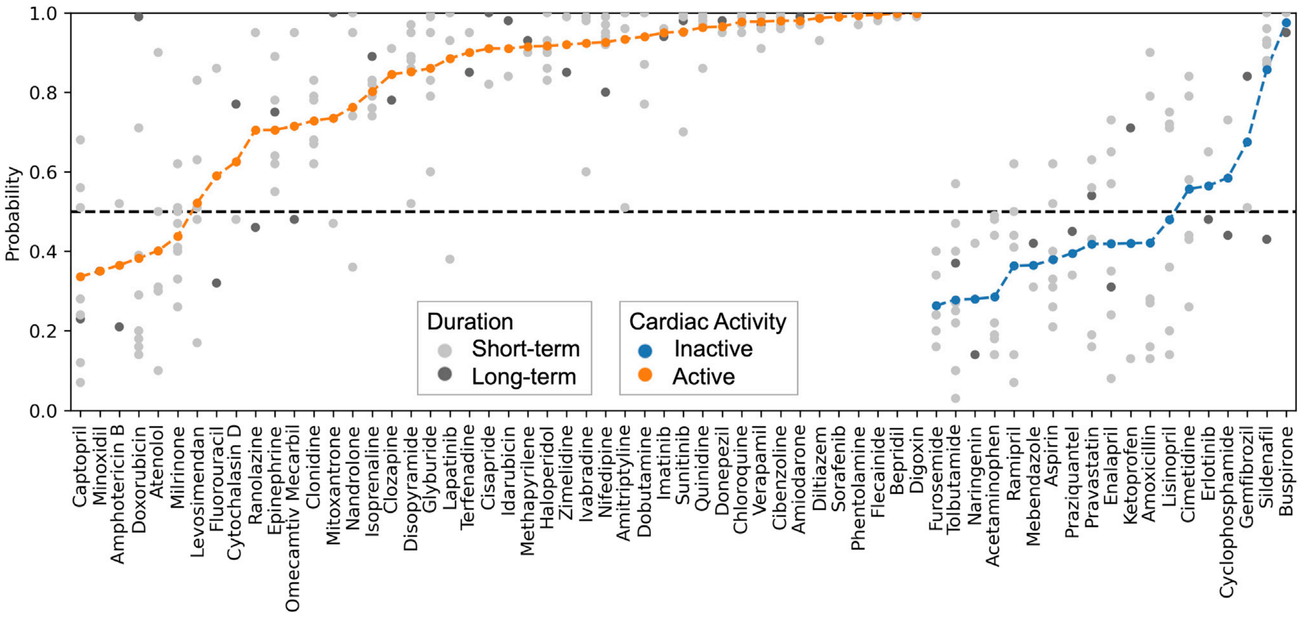

Figure 6. Predicted class probabilities of cardiac activity of compounds (as established via a leave-one-compound-out validation)
Multiple predictions for the same compound correspond to different replicates, where the gray points are short-term (30–60 min) samples and black points are long-term (72 h) samples. The colored points in the lines are the average probability of the compound being active. Orange means the compound is labeled by the FDA as cardiac active, while blue is cardiac inactive. Probability lower than 50% means the compound is predicted to be cardiac inactive by the machine learning model.

and to assess the ability to automatically flag cardiac-active compounds.

We first established the plausibility of the model by comparing the parameters derived using this toolkit and ScreenWorks and found that our toolkit is accurate in the derivation of parameters from the waveforms. Some mismatches mainly occurred in waveforms with multiple peaks. We also showed the plausibility by interpreting some parameters such as average intensity, the standard deviation of peak space, and the maximum of multi-peaks, which can be used to understand the shape of the waveforms.

Using correlation analysis, we next identified which parameters would be important for the prediction of clinical cardiac activity. The finding that amplitude-related parameters have the highest correlation is consistent with the common knowledge that changes in the intracellular concentration of calcium ions affect the contractile force, which is an important component in the pathogenesis of heart failure as well as rhythm changes (Thandroyen et al., 1991). Some parameters, including PW10 and the novel parameter shoulder/tail, have a higher correlation than PW90, which was highlighted by a previous study (Kopljar et al., 2018). However, a low correlation may not necessarily indicate irrelevance. For example, peak frequency has a correlation coefficient of only 0.1–0.2 with cardiac activity in different concentrations, which is lower than most of the other parameters, but we can still see a difference between positive controls and vehicle controls in terms of the distributions of this parameter.

Our results demonstrate that the prediction model using the derived parameters as features can predict the cardiac activities of compounds by using combinations of features better than using structural features (fingerprints) alone. Furthermore, it does so in an unbiased way, independent of manual curve fitting, making the data analysis workflow amenable to automation. The false predictions, on the other hand, give us hints for understanding the difference between calcium transient change and reported clinical activity. For example, although buspirone had a ground truth classification of inactive according to the FDA label, the model predicted it as being cardiac active. Observing the calcium transients, there is a noticeable change at high concentrations, and calcium transients cease at 50 μM (beat stop). We would therefore infer that the model has functioned as expected, with buspirone having an unexpected effect in this cellular model, at these concentrations, that is not reflected clinically.

Of course, the importance of pharmacokinetics (PK) is paramount when translating *in vitro* readouts to *in vivo* effects. In this regard, the selected standard concentration, 100 μM (or the highest achieved concentration below this), is far higher than the clinical C_{max}. This may lead, on the one hand, to false-positive predictions (e.g., for

cimetidine and erlotinib in Figure 6). On the other hand, the main objective of this work is not the prediction of *in vivo* effects at therapeutic concentrations. It is rather the evaluation of additional waveform parameters in a calcium transient readout, which has already been established for preclinical cardiac safety assessment, to determine both the utility of the parameters themselves and the ability to automate the parameter analysis workflow in an unbiased manner. The evaluation of molecules with the desired primary pharmacology can in this setup occur at higher throughput and before the PK properties of each molecule are established. This allows the opportunity to rank compounds based on changes in calcium transients and to support, e.g., design-make-test-analyze (DMTA) cycles in earlier stages of drug discovery in order to minimize the risk of later undesired cardiac activity.

Some of the false-negative predictions are not unexpected, because of the technical limitations of the *in vitro* hiPSC-CM assay regarding its capacity to reflect complex *in vivo* pharmacological mechanisms of cardiac-active compounds. One such consideration is that indirect modulators are not readily identified by the *in vitro* hiPSC-CM assay. For example, captopril is an angiotensin-converting enzyme (ACE) inhibitor that is primarily used to treat hypertension and congestive heart failure. Its cardiac activity stems from an effect on the renin-angiotensin system (RAS), rather than a direct effect on cardiomyocytes, and hence this effect is also not visible in the *in vitro* hiPSC-CM assay data used for our model, and more than half of the captopril waveform samples were predicted to be cardiac inactive (Figure 6). Similarly, amphotericin B leads to arrhythmias through electrolyte abnormalities, which are not reflected in calcium transients (Nix, 2014). Another consideration is that underlying *in vivo* physiological processes cannot be replicated in the *in vitro* hiPSC-CM assay. For example, atenolol, an antagonist of beta-adrenergic receptors, was not predicted to be cardiac active by our model. Atenolol has different effects in terms of calcium transients in different cell cultures, which would not be identified appropriately by the *in vitro* hiPSC-CMs assay employed here, a result that is also consistent with previous studies (Kopljar et al., 2018), where calcium transients were not significantly changed. However, a reduction of amplitude can be found in intrinsic cardiac adrenergic cells (Huang et al., 2005) and cardiac microtissues containing cardiomyocytes, cardiac microvascular endothelial cells, and cardiac fibroblast (Ravenscroft et al., 2016). This may indicate that baseline β-adrenergic stimulation (i.e., endogenous catecholamines) is required to detect the cardiac activity of atenolol or other beta-blockers, and hence co-culture systems containing cardiac endothelial cells might be more promising biological systems to use in this case, as they are able to synthesize and release catecholamines, such as adrenaline (Sorriento et al., 2012). A similar explanation may explain the results for milrinone, which induced no increase in the average amplitude of the calcium transients, given that enhancement in the baseline β-adrenergic stimulation is required for this compound to induce a positive inotropic action *in vitro* (Raffaeli et al., 1989).

There are several limitations in this study to be improved in the future. Some hyperparameters (thresholds) were introduced when deriving the parameters and they were empirically assigned based on our knowledge and the data we have. This work was performed on different batches of commercial hiPSC-CMs with basal peak frequencies equivalent to ∼1 Hz. For use with cellular sources with significantly higher or lower beating frequencies, further parameter optimization is likely required to fine-tune the analysis to the cellular system. As for the modeling, the training data have heterogeneous concentration settings, and as an approximation only one concentration was used instead of the entire concentration-response curve. In addition, exposure values were not taken into consideration in this study, which limits the application in the late stages of drug discovery (which, however, as described above, is not its primary purpose). Despite the good performance of the machine learning models in predicting the cardiac activities of the compounds, further validation of expanded chemical space and biological mechanisms is required to fully evaluate the potential of this approach.

In summary, we developed and evaluated a freely available Python toolkit to derive waveform parameters from the calcium transients of hiPSC-CMs. With a dataset of 63 compounds and their clinical cardiac activities based on FDA labels, we demonstrated the correlation between the derived parameters and the cardiac activity, and their predictive values. It is hoped that a combination of calcium transient detection in hiPSC-CMs, signaling analysis algorithms, and machine learning approaches will further enable the development of promising high-throughput platforms for the assessment of clinical cardiac activity in an unbiased, automated, and efficient manner.

EXPERIMENTAL PROCEDURES

Calcium transient waveform data

A detailed description of the experimental procedure to generate calcium transient waveform data has been given previously (Pointon et al., 2015). In brief, the hiPSC-CMs, cell culture thawing medium, and maintenance medium were purchased from Cellular Dynamics International (Madison, WI, USA). The hiPSC-CMs were cultured for 10 days (iCell maintenance media was refreshed every 48 h) prior to staning with the FLIPR Calcium 5 Assay Kit, a fluorescent dye, combined with the FLIPR Tetra

system was used to monitor changes in intracellular calcium ions (Sirenko et al., 2013). Cells were immediately transferred to a FLIPR Tetra and maintained at 37°C after the dyes were loaded. The fluorescence was evaluated at 480 nm excitation and 530 nm emission.

The raw data provided by AZ were the readout of the calcium flux, with 800 reads covering 100 s in duration, as well as 350 reads of baseline before compound addition. Each sample from GSK contained 600 reads covering 65.5 s in total, without baseline readout. Hence, the sampling interval for both data traces was about 100 ms, leading to each curve being approximated by about 10 data points at a beat frequency of 1 Hz. While this relatively low sampling frequency might not capture finer details of the waveforms, we still found it to be appropriate data to be used for the current purpose, given that it represents a standard readout used in the industry for the given objective and, hence, data available in a real-world situation. For each compound (across datasets) at least eight concentrations were tested, ranging from 0.32 nM to 300 μM. To avoid noise signals during detection, the raw data in all cases were smoothed via a 5-point quadratic polynomial Savitzky-Golay filter (Savitzky and Golay, 1964). To assist with the assessment of calcium transient waveform data from different wells and even from different plates over time, the data were scaled by subtracting the minimum relative fluorescence unit (RFU) count from each well from every point in that well.

Peak and subpeak detection

Peak detection was implemented by attempting to get the cycle number of each signal and to derive parameters from each cycle. SciPy (Virtanen et al., 2020) was used to identify the peaks of a waveform with their prominences. Peaks of which the prominence is lower than 20% or 10% of the maximum amplitude (MA) of the waveform would be regarded as false peaks. For the boundary of the waveform (the first and the last peaks), the prominences lie on the inner side of the peak, i.e., the leftmost peak bases only on the right side of the peak to measure the prominence.

Two approaches were used to identify multi-peaks: prominence based and tail based. In the prominence-based approach, if the prominence of a peak is lower than a threshold, and signal or amplitude is close to the last real peak within 10% of the MA, the peak will be regarded as a double peak or a subpeak. The threshold was determined empirically to be 50% of the maximum prominence (MP) when MA was lower than 250, and 70% of MP when MA was higher than 250. In some situations, the prominence-based approach cannot identify multi-peaks well. Based on our finding that cycles end with a long tail but the subpeaks do not, we recognized the subpeaks by comparing their tail with the maximum tail length in the waveform. After peak and subpeak detection, the signals of the whole waveform would be separated into several groups in terms of different cycles separated by peak points. Each group starts with a peak point (or the first point of the waveform) and ends by the last point before the next peak point (or the last point of the waveform), so each signal belongs to exactly one group. A cycle starts from a peak point and ends by the peak point of the next group. Normally the first and the last group would not be used to derive parameters because they are not a full cycle.

Quality control and normalization

Quality control was conducted based on peak frequency, average peak amplitude, and multi-peaks of the samples. First, if the peak frequency was zero, the sample would be removed from the dataset. Second, for the dataset from AZ, which has corresponding baseline waveforms (calcium transients before compound adding) available, samples were also removed in any of the following situations: (1) presence of a multiple peak, i.e., multi-peak is higher than 1, in the baseline waveform (indicating that the hiPSC-CMs are abnormal); (2) MA of the baseline waveform is lower than 100 (which would mean that the cells may have stopped beating); (3) there is a cycle where the key time points cannot be identified by our algorithm; or (4) peak frequency is outside ±20% of the median frequency of all DMSO samples within the same plate (which would indicate abnormal behavior of the sample). Then, for any plate from both datasets, if the vehicle controls present >20% robust coefficient of variation (RCV), the whole plate will be excluded. In this study, RCV was calculated based on the median absolute deviation.

Batch normalization was performed as follows. We first averaged the parameters of the vehicle control replicates in each plate to obtain the reference parameters, which were then subtracted from the values of treated samples in the same plate. Due to the different scales of value-type parameters, we then divided the subtracted parameters by the median value of the parameters of the vehicle control replicates to obtain the final normalized parameters (indicating a relative change of treated over control), while for deviation and ratio types, we only subtracted the vehicle control parameters without dividing.

Concentration selection

For each compound (and in both datasets provided), data for eight concentrations were available; however, those concentrations were not identical (see Table S3). We set a standard concentration set (0.01, 0.03, 0.1, 0.3, 1, 3, 10, 30, and 100 μM), and we matched concentrations used in experiments to the nearest concentration on this standard concentration set on a logarithmic scale. The sample with the lowest absolute difference on this scale was chosen; in case this was true for multiple samples, the higher absolute concentration was mapped to the respective standard concentration.

Correlation analysis and feature selection

The point-biserial correlation coefficient was used to evaluate the correlation between the parameters at different concentrations and the presence of the cardiac activity label as implemented in Python scripts (*scipy.stats.pointbiserialr*).

To understand the correlation among the waveform parameters themselves, the Pearson correlation was used as implemented in Python (*numpy.corrcoef*). Subsequently, hierarchical clustering (*scipy.cluster.hierarchy*) was applied to visualize the relationship between the parameters. Similarly defined parameters were removed to avoid redundancy and overfitting. Finally, we selected the 25 parameters with the highest overall point-biserial correlation coefficients at all concentrations for subsequent analysis. The names and definitions of the parameters selected in this way are listed in Table S1.

Data analysis and machine learning models

PCA was performed using scikit-learn (v.0.21.2) and all the samples were projected into a 2D plot (Pedregosa et al., 2011). The *sklearn.-decomposition.PCA* package centers the input data for each feature before implementing the single-value decomposition.

To evaluate the utility of the derived parameters for evaluating cardiac risk also in combination, we next trained random forest models on the derived parameters as implemented in scikit-learn. The number of trees was set to 100. Gini impurity was used as the criterion to measure the quality of a split. The other hyperparameters were set as default in scikit-learn.

The baseline model was built by random forest with molecular fingerprints. Extended connectivity fingerprints (ECFP) were calculated via RDKit (Bento et al., 2020). A radius of 2 was used, which is equivalent to ECFP4, and the fingerprints were folded into 2,048 bits, implemented by *GetMorganFingerprintAsBitVect.*

Model metrics, including sensitivity, precision, F1 score, and area under the receiver operating characteristic curve, were first calculated in a sample-wise manner, where each sample was regarded as one item in either the training set or the test set (leading to compounds with more replicates having a higher weight in the metrics). Furthermore, model metrics were also calculated in a compound-wise manner, where predictions of samples of the same compound would first be aggregated by averaging the probabilities of being cardiac active, so each compound would have an equal weight during the evaluation.

Data and code availability

The toolkit CardioWave is available as open source at https://github.com/zealseeker/CardioWave.

SUPPLEMENTAL INFORMATION

Supplemental information can be found online at https://doi.org/10.1016/j.stemcr.2022.01.009.

AUTHOR CONTRIBUTIONS

Y.H. developed the software, designed and conducted the experiments, and wrote the first version of the manuscript. S.W., P.A. and J.F. provided expert opinions on processing the calcium transient data and reviewed and revised the manuscript. O.O. joined the design of machine learning models and reviewed the manuscript. B.K., C.P., H.J., and S.G. reviewed and revised the manuscript. B.A. supervised the entire work and wrote the manuscript.

CONFLICT OF INTERESTS

S.W., F.J., B.K., C.P., and H.J. are employees of GlaxoSmithKline. P.A., O.O., and S.G. are employees of AstraZeneca.

ACKNOWLEDGMENTS

The research leading to the results has received support from the Cambridge Alliance on Medicines Safety (CAMS). The data were provided by AstraZeneca and GlaxoSmithKline.

Received: April 20, 2021
Revised: January 13, 2022
Accepted: January 13, 2022
Published: February 10, 2022

REFERENCES

Ahola, A., Pölönen, R.-P., Aalto-Setälä, K., and Hyttinen, J. (2018). Simultaneous measurement of contraction and calcium transients in stem cell derived cardiomyocytes. Ann. Biomed. Eng. *46*, 148–158.

Bedut, S., Seminatore-Nole, C., Lamamy, V., Caignard, S., Boutin, J.A., Nosjean, O., Stephan, J.-P., and Coge, F. (2016). High-throughput drug profiling with voltage- and calcium-sensitive fluorescent probes in human iPSC-derived cardiomyocytes. Am. J. Physiol. Heart Circ. Physiol. *311*, H44–H53.

Bento, A.P., Patrícia Bento, A., Hersey, A., Félix, E., Landrum, G., Gaulton, A., Atkinson, F., Bellis, L.J., De Veij, M., and Leach, A.R. (2020). An open source chemical structure curation pipeline using RDKit. J. Cheminform. *12*, 51.

Burden, N., Sewell, F., and Chapman, K. (2015). Testing chemical safety: what is needed to ensure the widespread application of non-animal approaches? PLoS Biol. *13*, e1002156.

Cook, D., Brown, D., Alexander, R., March, R., Morgan, P., Satterthwaite, G., and Pangalos, M.N. (2014). Lessons learned from the fate of AstraZeneca's drug pipeline: a five-dimensional framework. Nat. Rev. Drug Disco. *13*, 419–431.

Gintant, G., Sager, P.T., and Stockbridge, N. (2016). Evolution of strategies to improve preclinical cardiac safety testing. Nat. Rev. Drug Discov. *15*, 457–471.

Harmer, A.R., Abi-Gerges, N., Morton, M.J., Pullen, G.F., Valentin, J.P., and Pollard, C.E. (2012). Validation of an in vitro contractility assay using canine ventricular myocytes. Toxicol. Appl. Pharmacol. *260*, 162–172.

Hawkins, D.M. (2004). The problem of overfitting. J. Chem. Inf. Comput. Sci. *44*, 1–12.

Hoekstra, M., Mummery, C.L., Wilde, A.A.M., Bezzina, C.R., and Verkerk, A.O. (2012). Induced pluripotent stem cell derived cardiomyocytes as models for cardiac arrhythmias. Front. Physiol. *3*, 346.

Huang, M.-H., Bahl, J.J., Wu, Y., Hu, F., Larson, D.F., Roeske, W.R., and Ewy, G.A. (2005). Neuroendocrine properties of intrinsic cardiac adrenergic cells in fetal rat heart. Am. J. Physiol. Heart Circ. Physiol. *288*, H497–H503.

Hwang, H., Liu, R., Maxwell, J.T., Yang, J., and Xu, C. (2020). Machine learning identifies abnormal Ca2+ transients in human induced pluripotent stem cell-derived cardiomyocytes. Sci. Rep. *10*, 16977.

Juhola, M., Penttinen, K., Joutsijoki, H., Varpa, K., Saarikoski, J., Rasku, J., Siirtola, H., Iltanen, K., Laurikkala, J., Hyyrö, H., et al. (2015). Signal analysis and classification methods for the calcium transient data of stem cell-derived cardiomyocytes. Comput. Biol. Med. *61*, 1–7.

Kopljar, I., Lu, H.R., Van Ammel, K., Otava, M., Tekle, F., Teisman, A., and Gallacher, D.J. (2018). Development of a human iPSC

cardiomyocyte-based scoring system for cardiac hazard identification in early drug safety de-risking. Stem Cell Rep. *11*, 1365–1377.

Lu, H.R., Vlaminckx, E., Hermans, A.N., Rohrbacher, J., Van Ammel, K., Towart, R., Pugsley, M., and Gallacher, D.J. (2008). Predicting drug-induced changes in QT interval and arrhythmias: QT-shortening drugs point to gaps in the ICHS7B Guidelines. Br. J. Pharmacol. *154*, 1427–1438.

Lu, H.R., Zeng, H., Kettenhofen, R., Guo, L., Kopljar, I., van Ammel, K., Tekle, F., Teisman, A., Zhai, J., Clouse, H., et al. (2019). Assessing drug-induced long QT and proarrhythmic risk using human stem-cell-derived cardiomyocytes in a Ca2+ imaging assay: evaluation of 28 CiPA compounds at three test sites. Toxicol. Sci. *170*, 345–356.

Magdy, T., Schuldt, A.J.T., Wu, J.C., Bernstein, D., and Burridge, P.W. (2018). Human induced pluripotent stem cell (hiPSC)-Derived cells to assess drug cardiotoxicity: opportunities and problems. Annu. Rev. Pharmacol. Toxicol. *58*, 83–103.

Mellor, H.R., Bell, A.R., Valentin, J.-P., and Roberts, R.R.A. (2011). Cardiotoxicity associated with targeting kinase pathways in cancer. Toxicol. Sci. *120*, 14–32.

Molecular devices. https://www.moleculardevices.com/.

Nix, D.E. (2014). Cardiotoxicity induced by antifungal drugs. Curr. Fungal Infect. Rep. *8*, 129–138.

Octavia, Y., Tocchetti, C.G., Gabrielson, K.L., Janssens, S., Crijns, H.J., and Moens, A.L. (2012). Doxorubicin-induced cardiomyopathy: from molecular mechanisms to therapeutic strategies. J. Mol. Cell. Cardiol. *52*, 1213–1225.

Pedregosa, F., Varoquaux, G., Gramfort, A., Michel, V., Thirion, B., Grisel, O., Blondel, M., Prettenhofer, P., Weiss, R., Dubourg, V., et al. (2011). Scikit-learn: machine learning in Python. J. Mach. Learn. Res. *12*, 2825–2830.

Pointon, A., Harmer, A.R., Dale, I.L., Abi-Gerges, N., Bowes, J., Pollard, C., and Garside, H. (2015). Assessment of cardiomyocyte contraction in human-induced pluripotent stem cell-derived cardiomyocytes. Toxicol. Sci. *144*, 227–237.

Psaras, Y., Margara, F., Cicconet, M., Sparrow, A.J., Repetti, G., Schmid, M., Steeples, V., Willcox, J.A., Bueno-Orovio, A., Redwood, C., et al. (2021). CalTrack: high throughput automated calcium transient analysis in cardiomyocytes. Circ. Res. *129*, 326–341.

Racine, J.S. (2008). Nonparametric econometrics: a primer. Found. Trends Econ. *3*, 1–88.

Raffaeli, S., Ferroni, C., Spurgeon, H.A., and Capogrossi, M.C. (1989). Milrinone enhances cytosolic calcium transient and contraction in rat cardiac myocytes during beta-adrenergic stimulation. Int. J. Cardiol. *25*, S63–S69.

Ravenscroft, S.M., Pointon, A., Williams, A.W., Cross, M.J., and Sidaway, J.E. (2016). Cardiac non-myocyte cells show enhanced pharmacological function suggestive of contractile maturity in stem cell derived cardiomyocyte microtissues. Toxicol. Sci. *152*, 99–112.

Savitzky, A., and Golay, M.J.E. (1964). Smoothing and differentiation of data by simplified least squares procedures. Anal. Chem. *36*, 1627–1639.

Seabold, S., and Perktold, J. (2010). Statsmodels: econometric and statistical modeling with Python. In Proceedings of the 9th Python in Science Conference https://doi.org/10.25080/Majora-92bf1922-011.

Sirenko, O., Cromwell, E.F., Crittenden, C., Wignall, J.A., Wright, F.A., and Rusyn, I. (2013). Assessment of beating parameters in human induced pluripotent stem cells enables quantitative in vitro screening for cardiotoxicity. Toxicol. Appl. Pharmacol. *273*, 500–507.

Sorriento, D., Santulli, G., Del Giudice, C., Anastasio, A., Trimarco, B., and Iaccarino, G. (2012). Endothelial cells are able to synthesize and release catecholamines both in vitro and in vivo. Hypertension *60*, 129–136.

Spencer, C.I., Baba, S., Nakamura, K., Hua, E.A., Sears, M.A.F., Fu, C.-C., Zhang, J., Balijepalli, S., Tomoda, K., Hayashi, Y., et al. (2014). Calcium transients closely reflect prolonged action potentials in iPSC models of inherited cardiac arrhythmia. Stem Cell Rep. *3*, 269–281.

Thandroyen, F.T., Morris, A.C., Hagler, H.K., Ziman, B., Pai, L., Willerson, J.T., and Buja, L.M. (1991). Intracellular calcium transients and arrhythmia in isolated heart cells. Circ. Res. *69*, 810–819.

Virtanen, P., Gommers, R., Oliphant, T.E., Haberland, M., Reddy, T., Cournapeau, D., Burovski, E., Peterson, P., Weckesser, W., Bright, J., et al. (2020). SciPy 1.0: fundamental algorithms for scientific computing in Python. Nat. Methods *17*, 261–272.

Watanabe, H., Honda, Y., Deguchi, J., Yamada, T., and Bando, K. (2017). Usefulness of cardiotoxicity assessment using calcium transient in human induced pluripotent stem cell-derived cardiomyocytes. J. Toxicol. Sci. *42*, 519–527.

Wiśniowska, B., Mendyk, A., Fijorek, K., and Polak, S. (2014). Computer-based prediction of the drug proarrhythmic effect: problems, issues, known and suspected challenges. Europace *16*, 724–735.

Yang, Y.-Q., Cao, J.-L., Zou, H.-Y.-Y., Han, Z.-L., Sun, H.-W., Hou, M.-H., Chen, M.-L., Kong, X.-Q., and Lu, X.-Z. (2016). Effects of levosimendan on calcium transient in norepinephrine-cultured neonatal rat ventricular myocytes. Eur. Heart J. Suppl. *18*, A13–A20.

Yu, B., Ruan, M., Zhou, L., Xu, L., and Fang, T. (2012). Influence of cyclovirobuxine D on intracellular [Ca(2+)] regulation and the expression of the calcium cycling proteins in rat myocytes. Fitoterapia *83*, 1653–1665.

Stem Cell Reports, Volume *17*

Supplemental Information

Deriving waveform parameters from calcium transients in human iPSC-derived cardiomyocytes to predict cardiac activity with machine learning

Hongbin Yang, Will Stebbeds, Jo Francis, Amy Pointon, Olga Obrezanova, Kylie A. Beattie, Peter Clements, James S. Harvey, Graham F. Smith, and Andreas Bender

Figure S1. Occurrence of multi-peak of the 10 compounds with the highest occurrence.

The blue bars indicate the number of samples of the compound, and the green bars are the number of samples where at least one multiple-peak occurs. Only Ivabradine shows a significantly high occurrence compared to the background of negative control samples ($p<0.05$). Though the others are not significant, it is still possible that the multi-peak results from the effect of the compounds rather than the inherent variety of hiPSC-CMs.

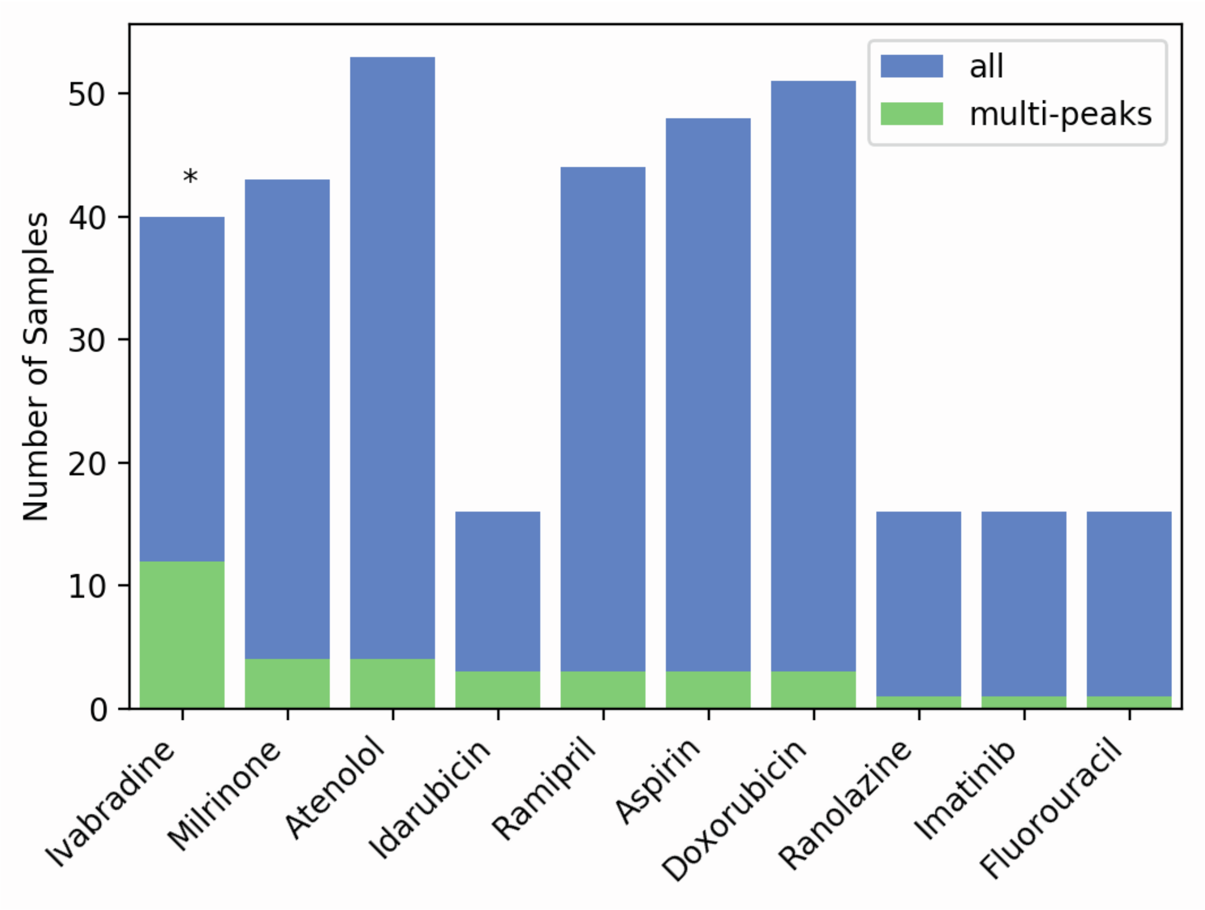

Figure S2. Samples with high Average Shoulder/Tail.

For replicates, only the highest Average Shoulder/Tail was kept and samples with Average Amplitude < 100 were removed since they are probably beat stop samples. It shows that almost all the samples with a high Average Shoulder/Tail (>0.5) are active, indicating that this parameter has very high precision in predicting cardiac activity. The only false positive, Buspirone, is also reasonable because it may have mild cytotoxicity under a high concentration (50 μM).

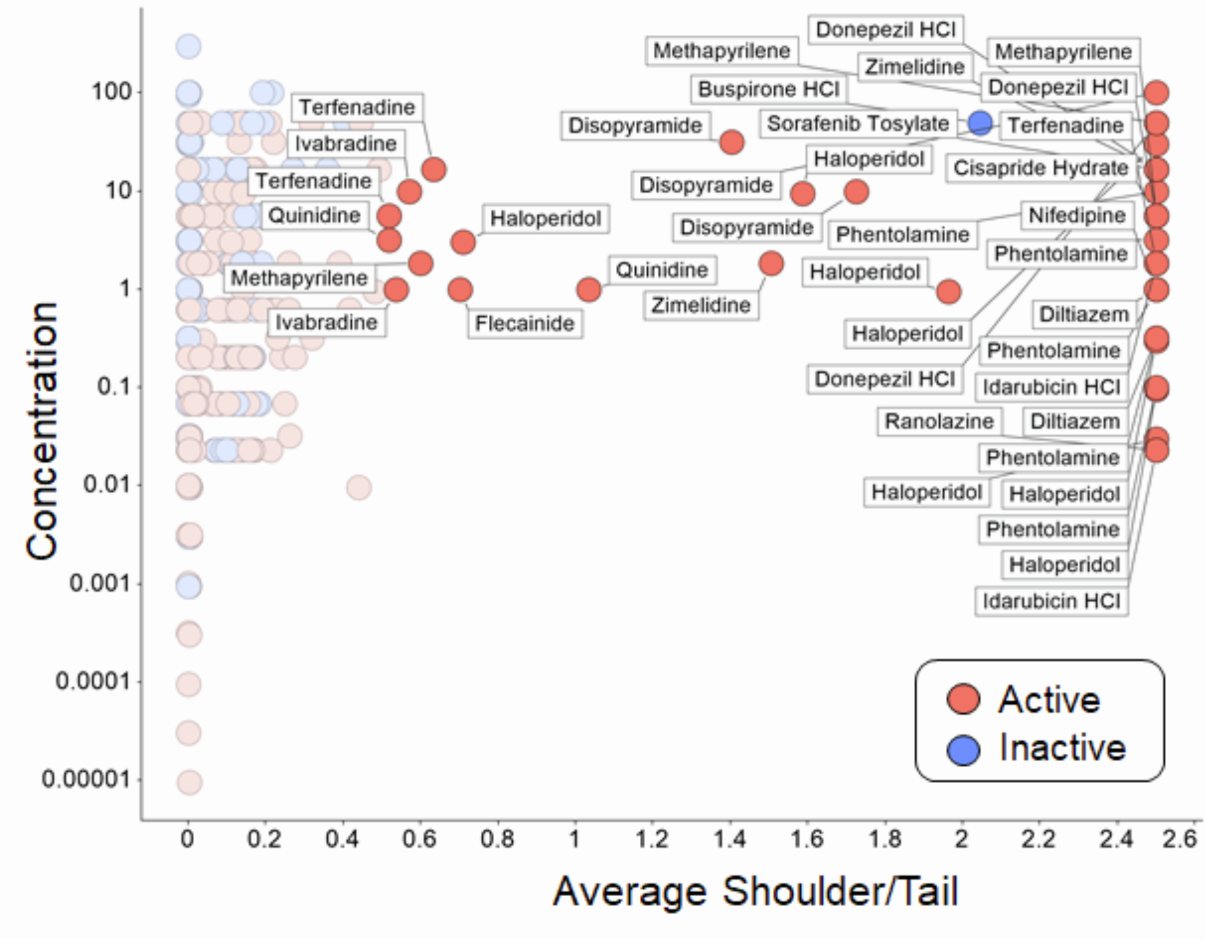

Figure S3. The sum of correlation coefficients between parameters and cardiac activities under different concentrations.

It can be seen that as the concentration increases, the correlation coefficients between the parameters and cardiac activity become higher in a general view.

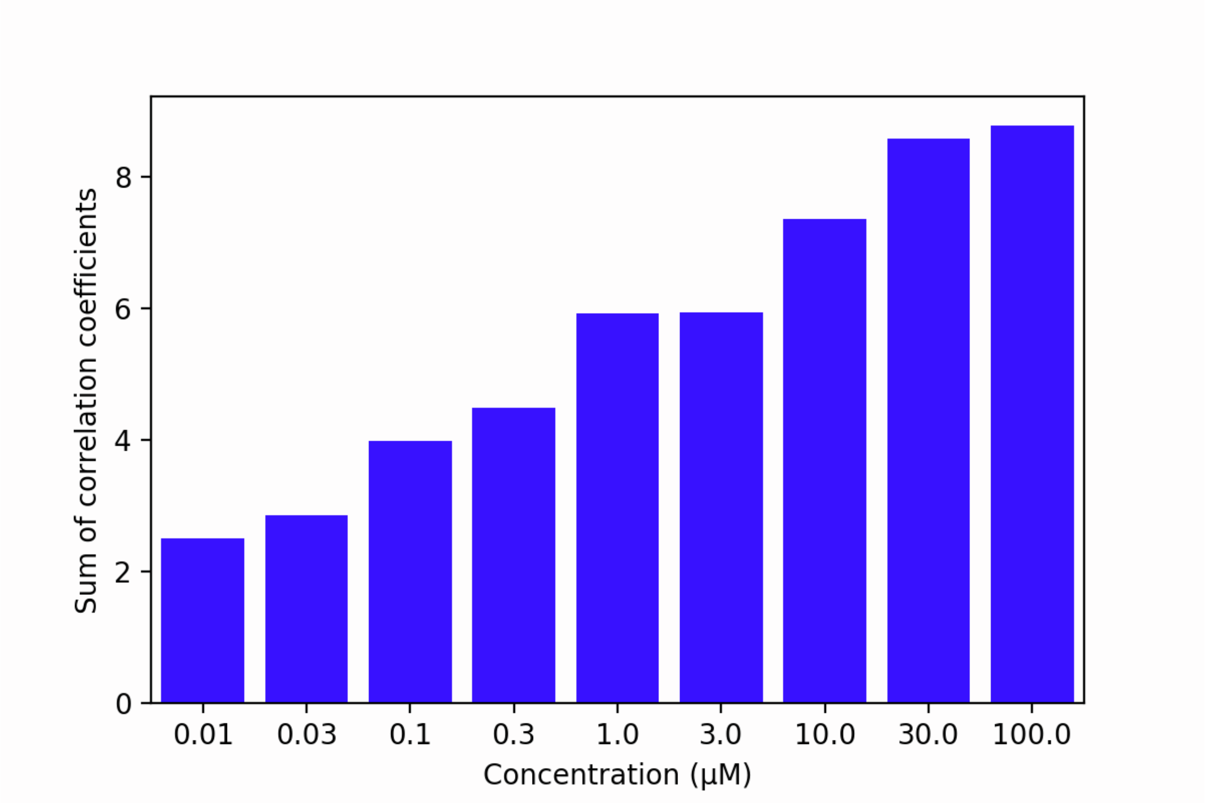

Figure S4. The Pearson coefficients between two parameters.

Generally, the coefficients are low except for some amplitude related parameters (RMS, Average Peak Amplitude and Average Intensity), indicating that these parameters can give much information to describe the waveform.

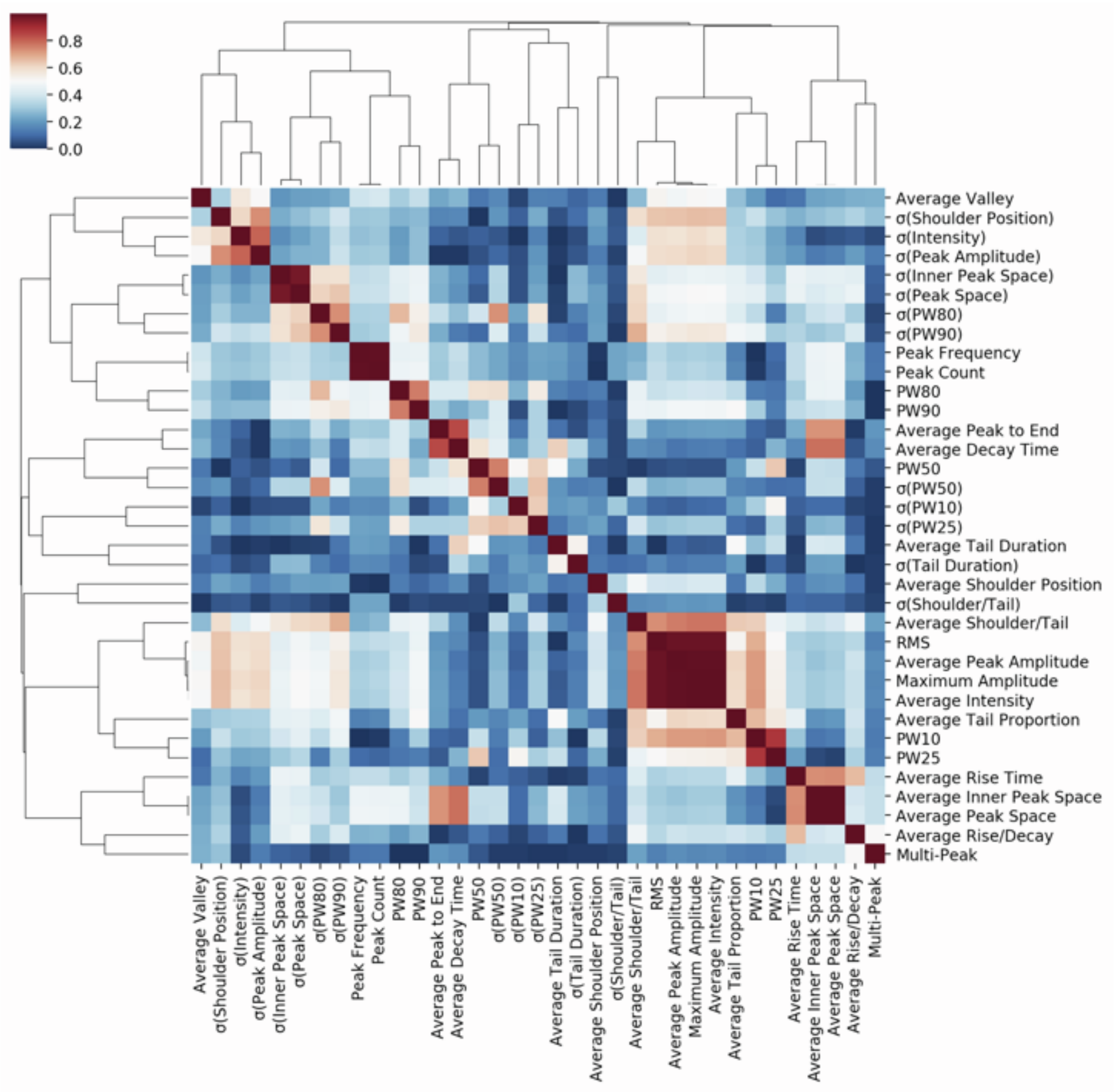

Figure S5. Distributions of the top 25 parameters ranked by the sum of the correlation between the parameters and the cardiac activity.

Samples are classified into three groups, vehicle (Neg) and positive (Pos) controls and other compounds (Treat). It can be seen that for most of the parameters, there is a clear difference between vehicle and negative controls and the treatment samples have overlapped with the positive and vehicle controls.

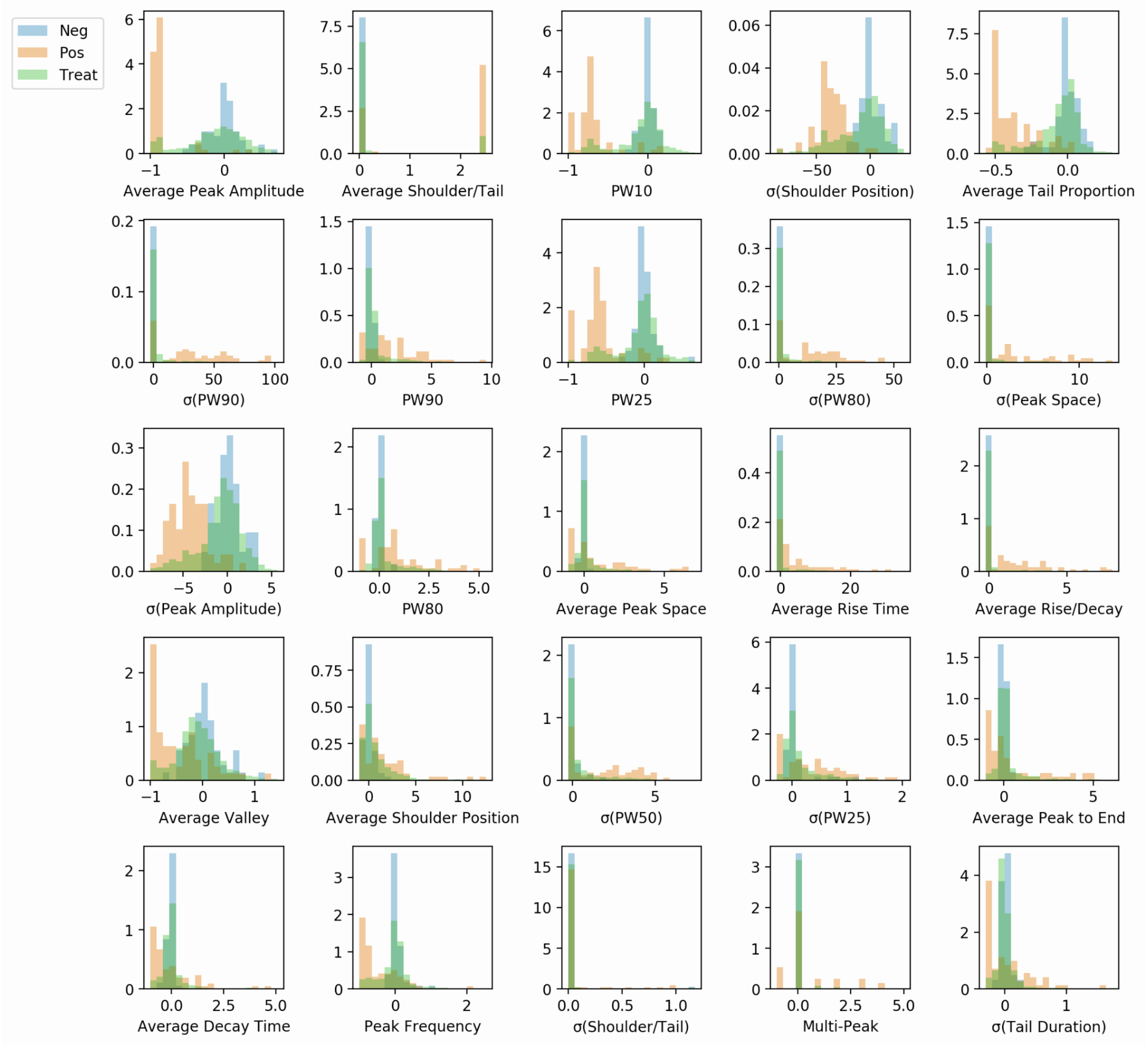

Figure S6. Principal component analysis of the top 25 parameters for active compounds.

The colour map is based on the logarithm of the concentrations, darker colours mean lower concentration. We can see that low concentration samples tend to be in the area which is overlapped with inactive compounds.

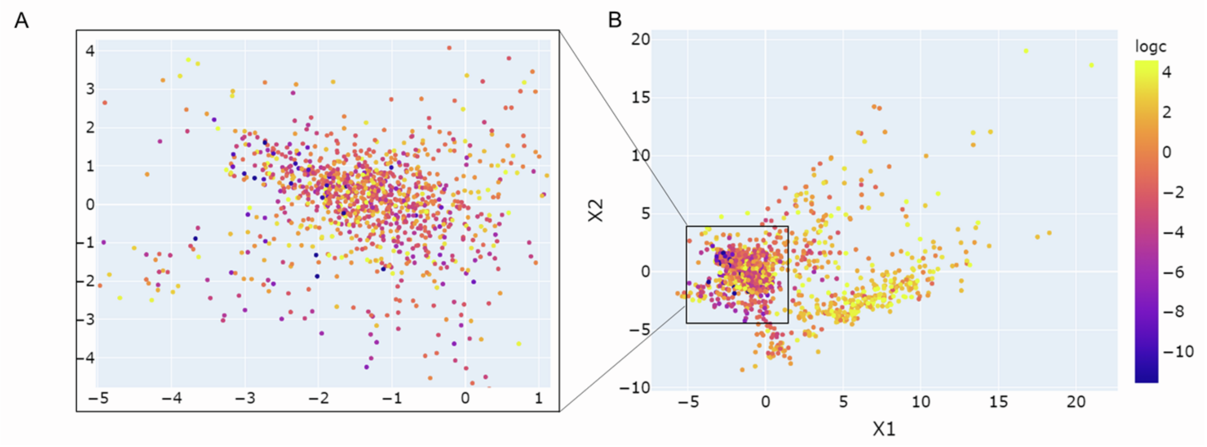

Figure S7. Distribution of distances between biological and technical replicates.

The distributions of the two datasets are almost the same to their mix, indicating that biological replicates and technical replicates present the same waveform varieties in terms of the parameters.

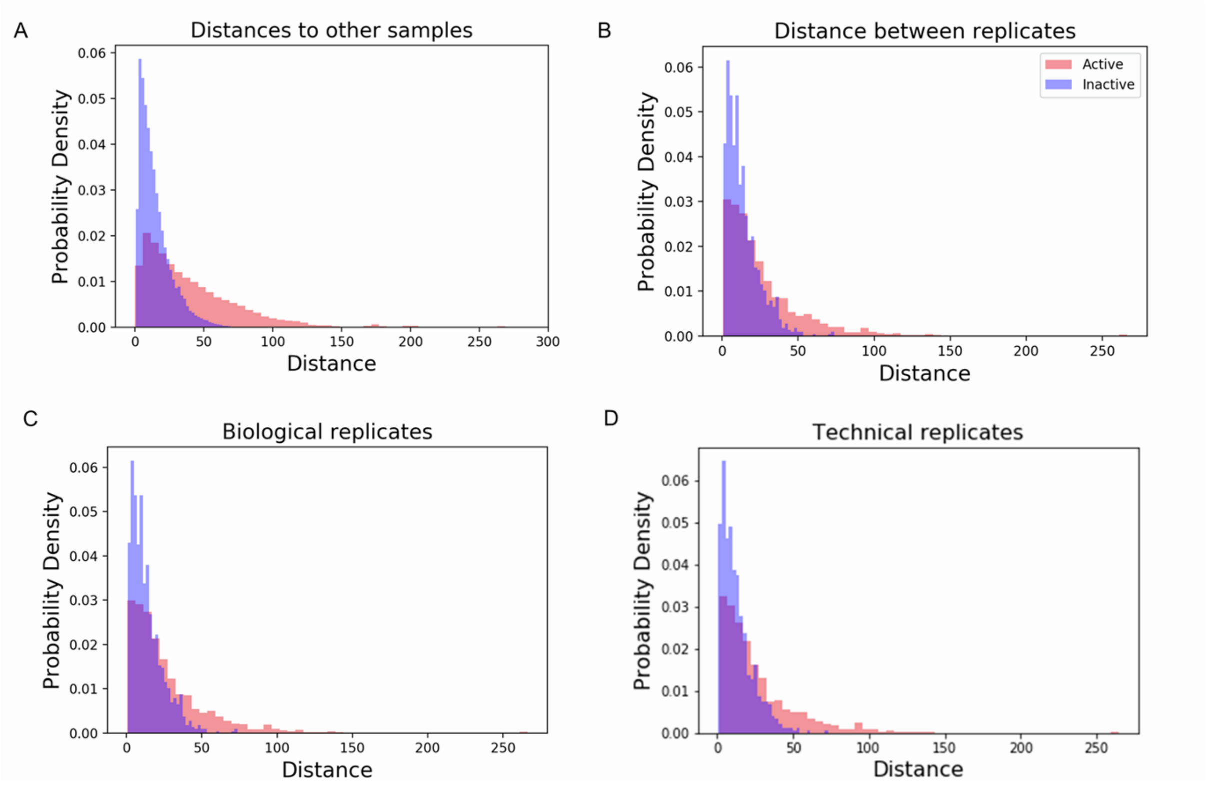

Table S1. Parameters that can be calculated by the toolkit.

Name	Variable	Type	Used in modelling	Annotation
Average Rise Time	up_length	Value	TRUE	Average rise time
Average Decay Time	down_length	Value	TRUE	Average duration of decreasing waveform, from peak point to the first point lower than valley value plus variance (10% of max)
Average Peak To End	full_down	Value	TRUE	Average of duration between peak and next starting point
Average Rise/Decay	rd_ratio	Ratio	TRUE	Average rise time/decay time ratio
Number of Peaks	n_peak	Value	FALSE	Number of peaks
Peak Frequency	freq	Value	TRUE	Number of period for the 100 seconds, 100/Peak Space
Maximum Amplitude	maximum	Value	FALSE	The highest amplitude
Average Peak Amplitude	avg_amplitude	Value	TRUE	Average peak amplitude
σ(Amplitude)	std_amplitude	Deviation	TRUE	The standard deviation of peak amplitudes
Average Intensity	avg_intensity	Value	FALSE	The average intensity (intensity is the highest signal in a cycle)
σ(Intensity)	std_intensity	Deviation	FALSE	The standard deviation of intensity
RMS	rms	Value	FALSE	Root mean square of the peak amplitudes
Average Peak Space	avg_lambda	Value	TRUE	The average duration of period
σ(Peak Space)	std_lambda	Deviation	TRUE	The standard deviation of period
Average Inner Peak Space	avg_inner_lambda	Value	FALSE	The average duration of periods removing outliers

σ(Inner Peak Space)	std_inner_lambda	Deviation	FALSE	The standard deviation of period removing outliers
Tail Proportion	tail_proportion	Ratio	TRUE	The average proportion of tail in one period
Average Tail Duration	avg_tail	Value	FALSE	The average duration of tail
Average Valley	avg_valley	Value	TRUE	The average intensity of valley points
Average Shoulder Position	avg_shoulder	Ratio	TRUE	The average relative position of shoulder in a period (0-1)
σ(Shoulder Position)	std_shoulder	Deviation	TRUE	The standard deviation of shoulder position
Average Shoulder/Tail	avg_shoulder_tail	Ratio	TRUE	The average ratio between prominence of shoulder and tail in a kernel density distribution
σ(Shoulder/Tail)	std_shoulder_tail	Deviation	TRUE	The standard deviation of shoulder tail ratio
Multi-Peaks	max_combo_peaks	Value	TRUE	Maximum of the number of peaks in one period
PWXX	PWXX_mean	Value	TRUE	The average of peak width at XX% from peak to bottom. XX is in {10, 25, 50, 80, 90}
σ(PWXX)	PWXX_std	Deviation	TRUE	Standard deviation of the peak widths
Uniformity	uniform	Binary	FALSE	Peak uniformity (p > 0.99 in KS test)
Noisy Waveform	noise	Binary	FALSE	If the waveform is noise, which means it is probabily a bad waveform
Double Peak	double_peak	Binary	FALSE	If there is double peak (max_combo_peaks > 1)
Fail	fail_analysis	Binary	FALSE	If there is a missing key point (starting point, down point etc.)

Used in modelling means whether the parameter(s) are used in machine learning models (25 in total)

Table S2. Numbers of samples and compounds used in this study

Data source	Compounds[1]	Test time point	Raw samples	Negative control samples	Positive control samples	Removed samples	Normalised samples
AstraZeneca	38	Baseline	2072	84	84	-	-
		0.5 hour	2072	59	69	390	1,554
GSK	36	1 hour	304	15	0	1	288
		72 hours	304	16	0	0	288
Sum	63		4752[2]	-	-	-	2,130

[1]Control compound (DMSO) is not counted in "compounds" column

[2]They are total number of waveforms, not the real samples

Table S3. Profile of the compounds

Compound name	Concentrations (μM)	Ground truth	Source	Duration
Amiodarone	0.023, 0.069, 0.206, 0.617, 1.852, 5.556, 16.667, 50	Active	GSK	1 hr, 72 hr
Amitriptyline	0.01, 0.03, 0.1, 0.3, 0.95, 3, 9.49, 30	Active	AZ	30min
Amoxicillin	0.1, 0.3, 0.95, 3.01, 9.51, 30.04, 94.94, 300	Inactive	AZ	30min
Amphotericin B	0.023, 0.069, 0.206, 0.617, 1.852, 5.556, 16.667, 50	Active	GSK	1 hr, 72 hr
Aspirin	0.03, 0.1, 0.32, 1.0, 3.17, 10.01, 31.65, 100	Inactive	AZ	30min
Atenolol	0.01, 0.03, 0.1, 0.3, 0.95, 3, 9.49, 30	Active	AZ	30min
Bepridil	0.003, 0.01, 0.03, 0.1, 0.32, 1.0, 3.16, 10	Active	AZ	30min
Buspirone	0.023, 0.069, 0.206, 0.617, 1.852, 5.556, 16.667, 50	Inactive	GSK	1 hr, 72 hr
Captopril	0.03, 0.1, 0.32, 1.0, 3.17, 10.01, 31.65, 100; 0.023, 0.069, 0.206, 0.617, 1.852, 5.556, 16.667, 50	Active	AZ/GSK	30min, 1 hr, 72 hr
Chloroquine	0.03, 0.1, 0.32, 1.0, 3.17, 10.01, 31.65, 100	Active	AZ	30min
Cibenzoline	0.03, 0.1, 0.32, 1.0, 3.17, 10.01, 31.65, 100	Active	AZ	30min
Cimetidine	0.03, 0.1, 0.32, 1.0, 3.17, 10.01, 31.65, 100	Inactive	AZ	30min
Cisapride	0.023, 0.069, 0.206, 0.617, 1.852, 5.556, 16.667, 50	Active	GSK	1 hr, 72 hr
Clonidine	0.01, 0.03, 0.1, 0.3, 0.95, 3, 9.49, 30	Active	AZ	30min
Clozapine	0.023, 0.069, 0.206, 0.617, 1.852, 5.556, 16.667, 50	Active	GSK	1 hr, 72 hr
Cyclophosphamide	0.023, 0.069, 0.206, 0.617, 1.852, 5.556, 16.667, 50	Inactive	GSK	1 hr, 72 hr
Cytochalasin D	0.023, 0.069, 0.206, 0.617, 1.852, 5.556, 16.667, 50	Active	GSK	1 hr, 72 hr
Digoxin	0.003, 0.01, 0.03, 0.1, 0.32, 1.0, 3.16, 10	Active	AZ	30min
Diltiazem	0.03, 0.1, 0.32, 1.0, 3.17, 10.01, 31.65, 100	Active	AZ	30min
Disopyramide	0.03, 0.1, 0.32, 1.0, 3.17, 10.01, 31.65, 100	Active	AZ	30min
Dobutamine	0.003, 0.01, 0.03, 0.1, 0.32, 1.0, 3.16, 10	Active	AZ	30min
Donepezil	0.023, 0.069, 0.206, 0.617, 1.852, 5.556, 16.667, 50	Active	GSK	1 hr, 72 hr
Doxorubicin	0.03, 0.1, 0.32, 1.0, 3.17, 10.01, 31.65, 100; 0.023, 0.069, 0.206, 0.617, 1.852, 5.556, 16.667, 50	Active	AZ/GSK	30min, 1 hr, 72 hr
Enalapril	0.03, 0.1, 0.32, 1.0, 3.17, 10.01, 31.65, 100; 0.023, 0.069, 0.206, 0.617, 1.852, 5.556, 16.667, 50	Inactive	AZ/GSK	30min, 1 hr, 72 hr
Epinephrine	0.00032, 0.001, 0.00317, 0.01, 0.0317, 0.1, 0.3165, 1; 0.023, 0.069, 0.206, 0.617, 1.852, 5.556, 16.667, 50	Active	AZ/GSK	30min, 1 hr, 72 hr
Erlotinib	0.023, 0.069, 0.206, 0.617, 1.852, 5.556, 16.667, 50	Inactive	GSK	1 hr, 72 hr
Flecainide	0.03, 0.1, 0.32, 1.0, 3.17, 10.01, 31.65, 100	Active	AZ	30min
Fluorouracil	0.023, 0.069, 0.206, 0.617, 1.852, 5.556, 16.667, 50	Active	GSK	1 hr, 72 hr

Furosemide	0.03, 0.1, 0.32, 1.0, 3.17, 10.01, 31.65, 100	Inactive	AZ	30min
Gemfibrozil	0.023, 0.069, 0.206, 0.617, 1.852, 5.556, 16.667, 50	Inactive	GSK	1 hr, 72 hr
Glyburide	0.03, 0.1, 0.32, 1.0, 3.17, 10.01, 31.65, 100	Active	AZ	30min
Haloperidol	0.01, 0.03, 0.1, 0.3, 0.95, 3, 9.49, 30	Active	AZ	30min
Idarubicin	0.023, 0.069, 0.206, 0.617, 1.852, 5.556, 16.667, 50	Active	GSK	1 hr, 72 hr
Imatinib	0.023, 0.069, 0.206, 0.617, 1.852, 5.556, 16.667, 50	Active	GSK	1 hr, 72 hr
Isoprenaline	0.00032, 0.001, 0.00317, 0.01, 0.0317, 0.1, 0.3165, 1; 0.023, 0.069, 0.206, 0.617, 1.852, 5.556, 16.667, 50	Active	AZ/GSK	30min, 1 hr, 72 hr
Ivabradine	0.03, 0.1, 0.32, 1.0, 3.17, 10.01, 31.65, 100	Active	AZ	30min
Ketoprofen	0.023, 0.069, 0.206, 0.617, 1.852, 5.556, 16.667, 50	Inactive	GSK	1 hr, 72 hr
Lapatinib	0.03, 0.1, 0.32, 1.0, 3.17, 10.01, 31.65, 100	Active	AZ	30min
Levosimendan	0.003, 0.01, 0.03, 0.1, 0.32, 1.0, 3.16, 10	Active	AZ	30min
Lisinopril	0.00095, 0.003, 0.0095, 0.3, 0.95, 3	Inactive	AZ	30min
Mebendazole	0.023, 0.069, 0.206, 0.617, 1.852, 5.556, 16.667, 50	Inactive	GSK	1 hr, 72 hr
Methapyrilene	0.023, 0.069, 0.206, 0.617, 1.852, 5.556, 16.667, 50	Active	GSK	1 hr, 72 hr
Milrinone	0.03, 0.1, 0.32, 1.0, 3.17, 10.01, 31.65, 100	Active	AZ	30min
Minoxidil	0.023, 0.069, 0.206, 0.617, 1.852, 5.556, 16.667, 50	Active	GSK	1 hr, 72 hr
Mitoxantrone	0.023, 0.069, 0.206, 0.617, 1.852, 5.556, 16.667, 50	Active	GSK	1 hr, 72 hr
Nandrolone	0.03, 0.1, 0.32, 1.0, 3.17, 10.01, 31.65, 100	Active	AZ	30min
Naringenin	0.023, 0.069, 0.206, 0.617, 1.852, 5.556, 16.667, 50	Inactive	GSK	1 hr, 72 hr
Nifedipine	0.003, 0.01, 0.03, 0.1, 0.32, 1.0, 3.16, 10; 0.023, 0.069, 0.206, 0.617, 1.852, 5.556, 16.667, 50	Active	AZ/GSK	30min, 1 hr, 72 hr
Omecamtiv Mecarbil	0.023, 0.069, 0.206, 0.617, 1.852, 5.556, 16.667, 50	Active	GSK	1 hr, 72 hr
Acetaminophen	0.03, 0.1, 0.32, 1.0, 3.17, 10.01, 31.65, 100	Inactive	AZ	30min
Phentolamine	0.03, 0.1, 0.32, 1.0, 3.17, 10.01, 31.65, 100	Active	AZ	30min
Pravastatin	0.03, 0.1, 0.32, 1.0, 3.17, 10.01, 31.65, 100; 0.023, 0.069, 0.206, 0.617, 1.852, 5.556, 16.667, 50	Inactive	AZ/GSK	30min, 1 hr, 72 hr
Praziquantel	0.023, 0.069, 0.206, 0.617, 1.852, 5.556, 16.667, 50	Inactive	GSK	1 hr, 72 hr
Quinidine	0.03, 0.1, 0.32, 1.0, 3.17, 10.01, 31.65, 100	Active	AZ	30min
Ramipril	0.03, 0.1, 0.32, 1.0, 3.17, 10.01, 31.65, 100	Inactive	AZ	30min
Ranitidine	0.03, 0.1, 0.32, 1.0, 3.17, 10.01, 31.65, 100	Inactive	AZ	30min
Ranolazine	0.023, 0.069, 0.206, 0.617, 1.852, 5.556, 16.667, 50	Active	GSK	1 hr, 72 hr
Sildenafil	0.01, 0.03, 0.1, 0.3, 0.95, 3, 9.49, 30; 0.023, 0.069, 0.206, 0.617, 1.852, 5.556, 16.667, 50	Inactive	AZ/GSK	30min, 1 hr, 72 hr

Sorafenib	0.023, 0.069, 0.206, 0.617, 1.852, 5.556, 16.667, 50	Active	GSK	1 hr, 72 hr
Sunitinib	0.01, 0.03, 0.1, 0.3, 0.95, 3, 9.49, 30; 0.023, 0.069, 0.206, 0.617, 1.852, 5.556, 16.667, 50	Active	AZ/GSK	30min, 1 hr, 72 hr
Terfenadine	0.023, 0.069, 0.206, 0.617, 1.852, 5.556, 16.667, 50	Active	GSK	1 hr, 72 hr
Tolbutamide	0.03, 0.1, 0.32, 1.0, 3.17, 10.01, 31.65, 100; 0.023, 0.069, 0.206, 0.617, 1.852, 5.556, 16.667, 50	Inactive	AZ/GSK	30min, 1 hr, 72 hr
Verapamil	0.003, 0.01, 0.03, 0.1, 0.32, 1.0, 3.16, 10; 0.023, 0.069, 0.206, 0.617, 1.852, 5.556, 16.667, 50	Active	AZ/GSK	30min, 1 hr, 72 hr
Zimelidine	0.023, 0.069, 0.206, 0.617, 1.852, 5.556, 16.667, 50	Active	GSK	1 hr, 72 hr

Table S4. Inter-correlation between parameters

Concentration (μM)	0.01	0.03	0.1	0.3	1	3	10	30	100
Average Peak Amplitude	0.093	0.127	0.242	0.219	0.321	0.338	0.453	0.486	0.475
Average Intensity	0.090	0.124	0.241	0.217	0.318	0.332	0.453	0.483	0.471
RMS	0.084	0.118	0.237	0.218	0.309	0.329	0.451	0.479	0.471
Maximum Amplitude	0.086	0.120	0.238	0.219	0.317	0.331	0.451	0.481	0.470
Average Shoulder/Tail	0.082	0.121	0.193	0.237	0.272	0.328	0.365	0.409	0.440
PW10	0.188	0.220	0.277	0.238	0.301	0.295	0.351	0.424	0.419
σ(Shoulder Position)	0.094	0.111	0.204	0.220	0.320	0.328	0.351	0.379	0.379
Average Tail Proportion	0.014	0.044	0.040	0.150	0.171	0.221	0.230	0.310	0.378
σ(PW90)	0.071	0.089	0.131	0.163	0.183	0.195	0.230	0.258	0.302
PW90	0.003	0.003	0.087	0.132	0.190	0.209	0.240	0.257	0.299
PW25	0.156	0.192	0.225	0.186	0.242	0.223	0.282	0.320	0.288
σ(PW80)	0.071	0.090	0.101	0.152	0.190	0.214	0.230	0.290	0.288
σ(Peak Space)	0.075	0.064	0.047	0.112	0.127	0.178	0.231	0.241	0.286
σ(Intensity)	0.024	0.003	0.071	0.033	0.115	0.141	0.213	0.301	0.282
σ(Amplitude)	0.025	0.047	0.090	0.053	0.126	0.090	0.264	0.276	0.281
σ(Inner Peak Space)	0.072	0.069	0.042	0.118	0.125	0.174	0.221	0.224	0.272
PW80	0.007	0.001	0.067	0.115	0.208	0.187	0.219	0.253	0.270
Average Peak Space	0.068	0.046	0.097	0.130	0.162	0.142	0.161	0.233	0.254
Average Inner Peak Space	0.066	0.043	0.095	0.130	0.161	0.140	0.157	0.227	0.248
Rise Time	0.049	0.028	0.042	0.084	0.109	0.084	0.101	0.234	0.236
Rise/Decay	0.050	0.036	0.073	0.081	0.122	0.118	0.157	0.197	0.216
Valley	0.061	0.035	0.055	0.028	0.139	0.126	0.255	0.203	0.198
Average Shoulder Position	0.178	0.180	0.148	0.193	0.188	0.121	0.044	0.058	0.176
σ(PW50)	0.125	0.147	0.185	0.204	0.231	0.250	0.321	0.314	0.174
σ(PW25)	0.140	0.159	0.151	0.122	0.194	0.161	0.191	0.222	0.173
Average Peak to End	0.012	0.024	0.085	0.123	0.159	0.159	0.155	0.123	0.160
Peak Count	0.054	0.094	0.059	0.068	0.025	0.007	0.067	0.129	0.151
Decay Time	0.045	0.036	0.098	0.106	0.129	0.141	0.157	0.132	0.151
Peak Frequency	0.051	0.093	0.057	0.071	0.018	0.001	0.056	0.120	0.142
σ(Shoulder/Tail)	0.107	0.140	0.143	0.118	0.149	0.127	0.085	0.191	0.130

Multi-Peak	0.082	0.065	0.012	0.033	0.046	0.033	0.012	0.105	0.121
σ(Tail Duration)	0.059	0.057	0.047	0.091	0.070	0.087	0.096	0.115	0.113
Average Tail Duration	0.037	0.014	0.062	0.067	0.094	0.089	0.098	0.063	0.039
PW50	0.024	0.039	0.040	0.014	0.068	0.044	0.004	0.014	0.021
σ(PW10)	0.067	0.072	0.011	0.051	0.024	0.001	0.014	0.034	0.009

Table S5. Performance of the machine learning model using leave-one-compound-out cross-validation

All data	Precision	Recall	F1 score	Accuracy	AUC
Sample-wise	0.84	0.85	0.84	0.79	0.84
Compound-wise	0.86	0.86	0.86	0.81	0.86
Simple model	0.82	0.84	0.83	0.76	0.83
Fingerprint-based model	0.72	0.95	0.82	0.71	0.6
Removing Inconsistent compounds					
Conventional method	0.97	0.76	0.85	0.81	-
Machine learning	0.88	0.9	0.89	0.85	-

Samples-wise means each sample is a data point when calculating metrics. Compound-wise means each compound is regarded as a data point by averaging the probability of different samples as a final output. The simple model was built by Number of Peaks and Average Peak Amplitude and assessed compound-wisely. The fingerprint-based model was built by molecular fingerprints (ECFP4) and random forest. The conventional method is based on concentration-response analysis using peak frequency and peak amplitudes. Inconsistent predictions between GSK and AZ were removed. The machine learning model is the same as the compound-wise one, but inconsistent compounds were removed during evaluation for a fair comparison.
